# Discovery of repurposed drugs that disrupt intracellular replication of *Burkholderia pseudomallei* and *Burkholderia mallei* and protect against lethal aerosol infection

**DOI:** 10.1371/journal.ppat.1014596

**Published:** 2026-09-16

**Authors:** Nichole Orr-Burks, Sarah R. Hosking, Steven P. Maher, Dennis E. Kyle, Malina A. Bakowski, Robert J. Hogan, Eric R. Lafontaine

**Affiliations:** 1 Department of Biomedical Sciences, College of Veterinary Medicine, University of Georgia, Athens, Georgia, United States of America; 2 Department of Infectious Diseases, College of Veterinary Medicine, University of Georgia, Athens, Georgia, United States of America; 3 Center for Tropical and Emerging Global Diseases, University of Georgia, Athens, Georgia, United States of America; 4 Department of Cellular Biology, University of Georgia, Athens, Georgia, United States of America; 5 Division of Scripps Research, Calibr-Skaggs Institute for Innovative Medicines, La Jolla, California, United States of America; The University of North Carolina at Chapel Hill School of Medicine, UNITED STATES OF AMERICA

## Abstract

*Burkholderia pseudomallei (Bp)* and *Burkholderia mallei (Bm)* are the etiological agents of the fatal diseases melioidosis and glanders, respectively. No licensed vaccine is available to protect against these facultative intracellular bacteria and treatment of infection is difficult, requiring intensive and prolonged antimicrobial therapy with low success rates due to intrinsic resistance of *Bp* and *Bm* to most antibiotics. This, combined with concerns regarding their adversarial use as biological warfare agents, emphasize an immediate need to identify novel and effective countermeasures for the organisms. Herein, we developed an *in vitro* high-content imaging, high-throughput screening platform and tested a **L**ibrary of **P**harmacologically **A**ctive **C**ompounds (LOPAC) and the Repurposing, **F**ocused **R**escue, and **A**ccelerated **ME**dchem (ReFRAME) library of small molecules for bioactive compounds protecting against bacterial intracellular replication, which is a key pathogenicity trait of *Bp* and *Bm.* We first analyzed confocal microscopy images of *Burkholderia*-infected monolayers of Vero E6 cells and established a reproducible endpoint readout that quantifies cytopathic effects and the formation of plaques during infection. Following this, we screened ~14,800 small molecules and identified 170 unique structures providing ≥  75% inhibition of intracellular replication of *Bp* and *Bm* with EC_50_ values ranging from 1 pM to 9.99 µM. Of these hits, 23 were found to disrupt the intracellular replication of both organisms including 8 compounds targeting dihydrofolate reductase, 3 tetracycline-class antibiotics, an inhibitor of bacterial leucyl-tRNA synthetase (epetraborole), and 2 host-directed compounds targeting receptors on the surface of mammalian cells. Selected compounds were evaluated *in vivo* using a BALB/c mouse model of aerosol infection and we discovered that daily treatment with epetraborole (20 mg/kg) and the fluoroquinolone levofloxacin (5 mg/kg) completely protected mice against exposure to lethal doses of *Bm* and *Bp*, reducing bacterial burdens in the lungs and spleen to undetectable levels in all animals by day 14 post-challenge.

## Author summary

Pathogens that can survive inside host cells such as *Burkholderia pseudomallei* and *Burkholderia mallei* are difficult to treat because they damage host cells from within and are resistant to many conventional antimicrobial therapies. We developed an *in vitro* high-throughput, high‑content imaging platform that allows us to monitor this process and measure how well different compounds protect infected cells. Using this system, we screened nearly 14,800 pharmacologically active molecules. Many caused a measurable reduction in cell damage, revealing both known antibiotic classes and host‑directed compounds with activity against intracellular *Burkholderia*. We selected a small subset of candidates for *in vivo* testing and found that epetraborole and levofloxacin provided full protection against both *Burkholderia* species in a mouse aerosol infection model, eliminating detectable bacteria from target organs. Our study shows how image‑based phenotypic screening can rapidly identify new therapeutic opportunities, including drugs already available for other uses. This approach may help accelerate treatment discovery for pathogens that depend on intracellular survival and cause severe disease.

## Introduction

*Burkholderia pseudomallei* (*Bp*) and *Burkholderia mallei* (*Bm*) are closely-related bacteria causing fatal infections in humans and animals. *Bp* is found in soil, is endemic in ~ 45 countries bordering the equator, can infect most mammals, and causes the emerging disease melioidosis in humans. Data suggest > 165,000 people are infected every year with *Bp* resulting in over 89,000 fatalities. Infections are notoriously underreported as bacterial culture is the primary method of diagnosis. This, combined with intrinsic resistance to antibiotics, places melioidosis as a leading cause of mortality amongst tropical diseases [1–6]. *Bm* evolved directly from *Bp* but is host-adapted to persist only in equids, causing the highly contagious and fatal zoonosis glanders. The disease is endemic in Asia, the Middle East, and South America, and is closely monitored by the World Organization for Animal Health as glanders is considered a re-emerging biosafety and biosecurity threat due to its prior documented use in biological warfare [7–11]. The U.S. Federal Select Agent Program classifies *Bp* and *Bm* as Tier 1 agents (highest threat level) and research with these wild-type organisms must be performed in a BSL-3 laboratory under stringent regulations and practices to prevent release, misuse and infection.

The diseases caused by *Bp* and *Bm* share many clinical and pathological features, including the ability to cause life-threatening pneumonia and septicemia, as well as the capacity for intracellular survival, actin-based motility, and cell-to-cell spread leading to multinucleated giant cells and abscess formation. However, important differences exist. *Bp* is an environmental saprophyte endemic to soil and water in tropical regions, capable of infecting a broad range of mammals, whereas *Bm* is a host-adapted clone of *Bp* that primarily persists in equids, exhibits reduced environmental survival and is considered a re-emerging biosecurity threat due to its prior use in biological warfare. These ecological and host-range differences influence their respective transmission dynamics, geographic distribution, and regulatory oversight, while both remain classified as Tier 1 Select Agents due to their high virulence and potential for adversarial use. In humans, infection generally occurs via aerosols or skin abrasions and manifests as life-threatening pneumonia and/or septicemia. Pathogenesis is complex and involves the coordinated expression of many virulence factors that support extracellular and intracellular replication of bacteria, seeding of deep tissues, and host persistence [12–16]. The organisms’ ability to thrive intracellularly is a key virulence trait promoting persistence in target organs (liver, lungs, spleen, lymph nodes) where bacteria form granulomas and abscesses that are difficult to treat and clear. No licensed vaccine exists to protect humans or animals, and treatment options are limited due to resistance to most antibiotics. Current front-line antimicrobials include intravenous ceftazidime or meropenem for 2–8 weeks followed by oral trimethoprim-sulfamethoxazole or amoxicillin/clavulanic acid for 3–6 months [17–19]. Even with timely diagnosis and aggressive antibiotic therapy, response is slow and complete eradication of *Bp* and *Bm* from target organs is difficult to achieve even with surgical intervention. Patients experience recrudescence and high mortality rates (19–51% for melioidosis, ≥ 50% for glanders). Hence, melioidosis and glanders represent significant global biosecurity and public health problems and the development of novel countermeasures is a critical unmet need.

*Bp* and *Bm* can invade, survive and replicate within many types of host cells including professional phagocytes and epithelial cells. The organisms use Type 3 and Type 6 secretion systems to inject effector proteins inside host cells and subvert eukaryotic cellular functions. Once internalized, *Bp* and *Bm* escape endocytic vacuoles, enter the cytoplasm, replicate, and use actin-based motility to spread to neighboring cells by a process involving the fusion of host plasma membranes to create a conduit between cytosolic compartments. The intercellular spread of organisms results in the formation of large multinucleated giant cells (MNGCs) by professional phagocytes and syncytia by epithelial cells, which eventually lyse to form plaques in cell culture assays. *In vivo*, MNGCs and syncytia development are major pathological hallmarks of *Bp* and *Bm* infection driving bacterial dissemination, immune evasion, and persistence [5,7,13,16,20,21].

With this unmet medical need in mind, we developed an *in vitro* high-throughput screening platform for the discovery of small molecules that disrupt intracellular replication of *Bp* and *Bm*. The method, which is compatible with BSL-3 containment practices, leverages high-content imaging data to monitor key phenotypic outcomes including reduction in the formation of MNGCs and intracellular replication of bacteria. We utilized the platform to screen *n* = 14,758 compounds from two repurposing libraries and identified several structures with low toxicity to host cells and robust bioactivity against *Bp*, *Bm* and the closely-related bacterium *Burkholderia thailandensis*. We evaluated the therapeutic efficacy of selected leads *in vivo* using a BALB/c mouse model of aerosol infection and show that two repurposed drugs protect against exposure to lethal doses of *Bp* and *Bm*, reduce bacterial burdens in the lungs, and decrease dissemination to deep tissues.

## Results

### Optimized 384-well infection assay leads to reproducible high-content imaging endpoint readouts and the identification of bioactive compounds

The BSL-2 organism *Burkholderia thailandensis (Bt)* strain DW503 was initially used to develop a high-content imaging, high-throughput screening platform for the discovery of compounds that protect against intracellular bacterial replication. Strain DW503 is a well-characterized surrogate of *Bp* and *Bm* and commonly used to study the interactions of *Burkholderia spp.* with host cells [22,23] because it recapitulates key intracellular pathogenic behaviors—including host cell invasion, intracellular replication, actin-based motility, and cell-to-cell spread—while remaining safe to handle outside of BSL-3 containment. This surrogate allowed efficient assay optimization and validation prior to transitioning the platform to wild-type *Bp* and *Bm* under BSL-3 conditions. The African Green Monkey kidney epithelial cell line Vero E6, a staple of high throughput screening and toxicology research, was selected to establish the infection assay in 384-well plates. Herein, we tested variables including bacterial inoculum (volumes and CFU), host cell seeding densities, incubation times for bacterial attachment and entry into cells, kanamycin overlay concentrations to kill extracellular bacteria, and incubation periods to allow intracellular *Burkholderia* to replicate and form plaques. Vero E6 cells were seeded into 384-well plates with a Biomek NXP automated workstation. The inocula were prepared by suspending plate-grown bacteria to an optical density of 1.0 at a wavelength of 600 nm (OD_600_ of 1.0, 10^8^ CFU/mL), serially diluting the suspension, and back-titering to verify the number of organisms used to infect monolayers. Following infection, liquid medium overlay containing kanamycin was dispensed into the wells of cell culture plates with an epMotion 96 semi-automated electronic pipette. At experimental endpoints, the 384-well plates were treated with fixative containing Hoechst dye to stain the nuclei of Vero E6 cells and analyzed using an ImageXpress Micro Confocal High-Content Imaging System and a MetaXpress software. CDD Vault was used for the storage and management of data.

**Fig 1:**
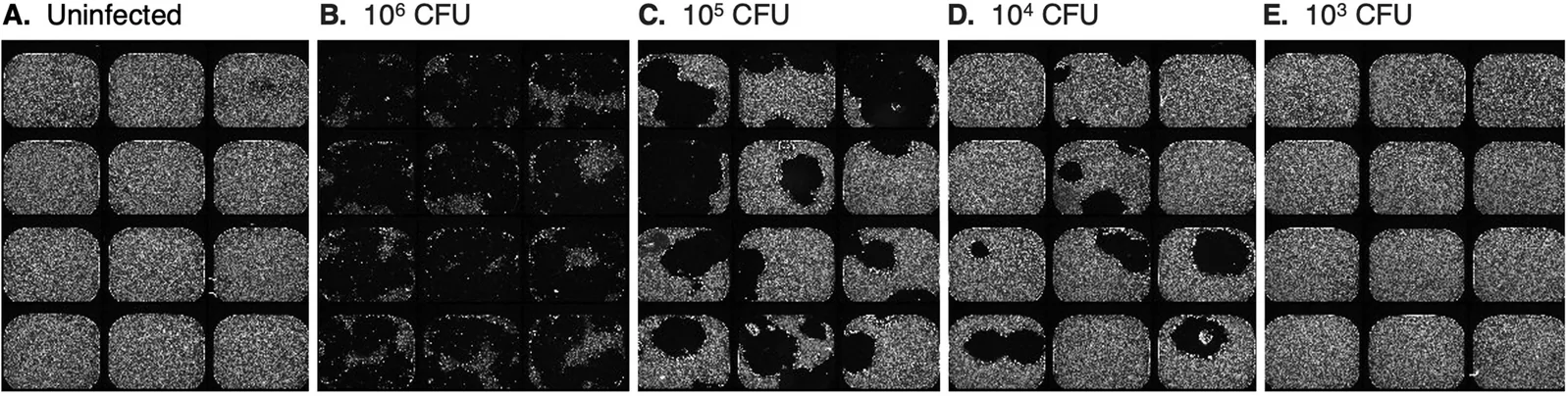
Development and optimization of 384-well infection assay using *Burkholderia thailandensis* strain DW503. Vero E6 cells (6,500 cells/well) seeded in 384-well plates were mock-infected with 10 µL of PBS (panel A) or infected with 10 µL of bacterial suspensions containing 10^6^ (panel B), 10^5^ (panel C), 10^4^ (panel D) or 10^3^ (panel E) colony forming units (CFU) of *Bt* DW503. Following this, the monolayers were incubated at 37^o^C for 3 hours, treated with medium containing 100 µg/mL of kanamycin, incubated for 48 hours at 37^o^C to allow the formation of plaques, treated with fixative containing Hoechst dye, and analyzed using an ImageXpress Micro Confocal High Content Imaging System. Representative images are shown. During optimization, wells were also scored visually for the presence of overwhelming extracellular bacterial growth, defined as > 25% premature monolayer destruction or obscuration of the monolayer by bacteria in the medium.

To begin optimizing the platform, infection volumes of 5 and 10 µL were first evaluated using the *Bt* DW503 suspension at OD_600_ of 1.0 and the 10^-1^, 10^-2^, and 10^-3^ dilutions of the suspended bacteria; the seeding density of host cells was kept constant at 6,500 cells per well. Once inoculated, the 384-well plates were incubated at 37^o^C for 1 or 3 hours to allow bacterial attachment and entry into Vero E6 cells. Following this, the infected monolayers were overlayed with medium containing 100 or 500 µg/mL (final concentration) of the antibiotic kanamycin to kill extracellular bacteria and incubated at 37^o^C for a period of 48 or 72 hours to allow replication of intracellular *Burkholderia* and the formation of plaques. Wells were visually inspected daily under an inverted light microscope, and a subjective scoring system was applied to assess extracellular bacterial growth. “Overwhelming” extracellular growth was defined as wells showing > 25% monolayer destruction prior to the 48-hour endpoint or wells in which extracellular bacteria were so abundant that the monolayer was no longer clearly discernible. We acknowledge that complete elimination of all extracellular bacteria is not possible under these conditions. However, the selected kanamycin concentrations effectively suppressed extracellular growth while allowing intracellular replication and plaque formation, thereby enriching for phenotypes driven by intracellular bacterial activity. Fig 1 shows representative high-content confocal microscopy images of these optimization experiments. We found that infecting cells with 5 or 10 µL of the undiluted *Bt* DW503 suspension (10^6^ CFU, Fig 1B) resulted in the near complete destruction of monolayers and overwhelming extracellular bacterial growth in the overlay medium under all conditions tested, while infection with 5 or 10 µL of the 10^-3^ dilution (10^3^ CFU, Fig 1E) did not yield plaques. Infection with 5 or 10 µL of the 10^-2^ dilution (10^4^ CFU, Fig 1D) and with 5 µL of the 10^-1^ dilution (10^5^ CFU) produced plaques without visible extracellular bacterial growth, but plaques were not present in all wells. By increasing the infection volume from 5 µL of the 10^-1^ dilution to 10 µL of the 10^-1^ dilution (10^5^ CFU, Fig 1C) we were able to produce plaques without visible extracellular bacterial growth consistently in all wells. Only data from the 10 µL volumes are shown in Fig 1 for simplicity. Collectively, infecting with 10 µL of the 10^-1^ dilution (10^5^ CFU), incubating 3 hours to allow bacterial attachment and entry, treating with medium containing 100 ug/mL of kanamycin, and a plaque formation period of 48 hours provided the best results and reproducibly yielded countable plaques in all wells without visible *Burkholderia* in the overlay medium.

**Fig 2:**
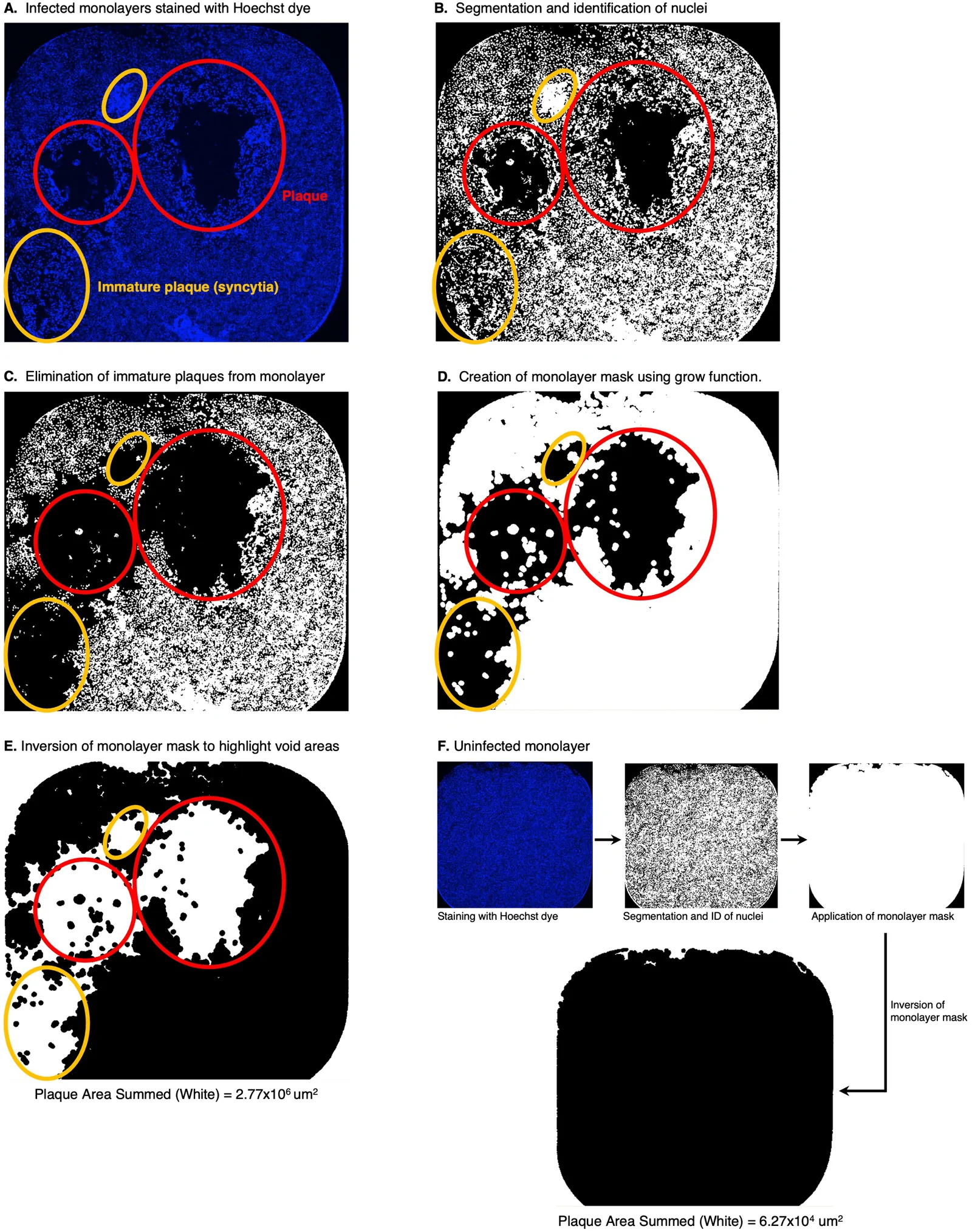
Development of filters and masks to analyze high-content images of monolayers infected with *Burkholderia thailandensis* strain DW503. Vero E6 cells (6,500 cells/well) seeded in 384-well plates were infected with 10 µL of bacterial suspension containing 10^5^ CFU, incubated 3 hours at 37^o^C to allow bacterial attachment and entry, overlayed with medium containing 100 µg/mL of kanamycin, incubated at 37^o^C for 48 hours to allow the formation of plaques, treated with fixative containing Hoechst dye, and analyzed using an ImageXpress Micro Confocal High Content Imaging System and MetaXpress software. Panels A-E show the use of filters and masks to analyze infected cells and the calculation of a Plaque Area Summed endpoint (PAS) readout value, which corresponds to the accrued area of plaque formation in µm^2^. Panel F illustrates the analysis of uninfected cells. Representative images are shown.

High-content confocal microscopy images were analyzed with the MetaXpress software to measure host cell damage during infection and establish an endpoint readout for the assay. As illustrated in Figs 2A-2E, both clear plaques (red circles) and syncytia (*i.e.,* immature plaques, yellow circles) were used to define the endpoint. First, all stained nuclei were identified, and a fluorescence intensity threshold was applied to eliminate the background signal and emphasize clear plaques (Fig 2B). Next, syncytia were delineated based on the higher average fluorescence intensity of their nuclei and removed from view (Fig 2C). Following this, we used the MetaXpress software’s pixel grow function to create an intact monolayer mask that encompasses cellular areas beyond the stained nuclei (Fig 2D). Lastly, the intact monolayer mask was inverted to highlight void plaque areas in white and to calculate an endpoint numerical value for the assay, which we termed **P**laque **A**rea **S**ummed (PAS, Fig 2E). Fig 2F illustrates the use of filters and masks to analyze uninfected cells.

**Fig 3:**
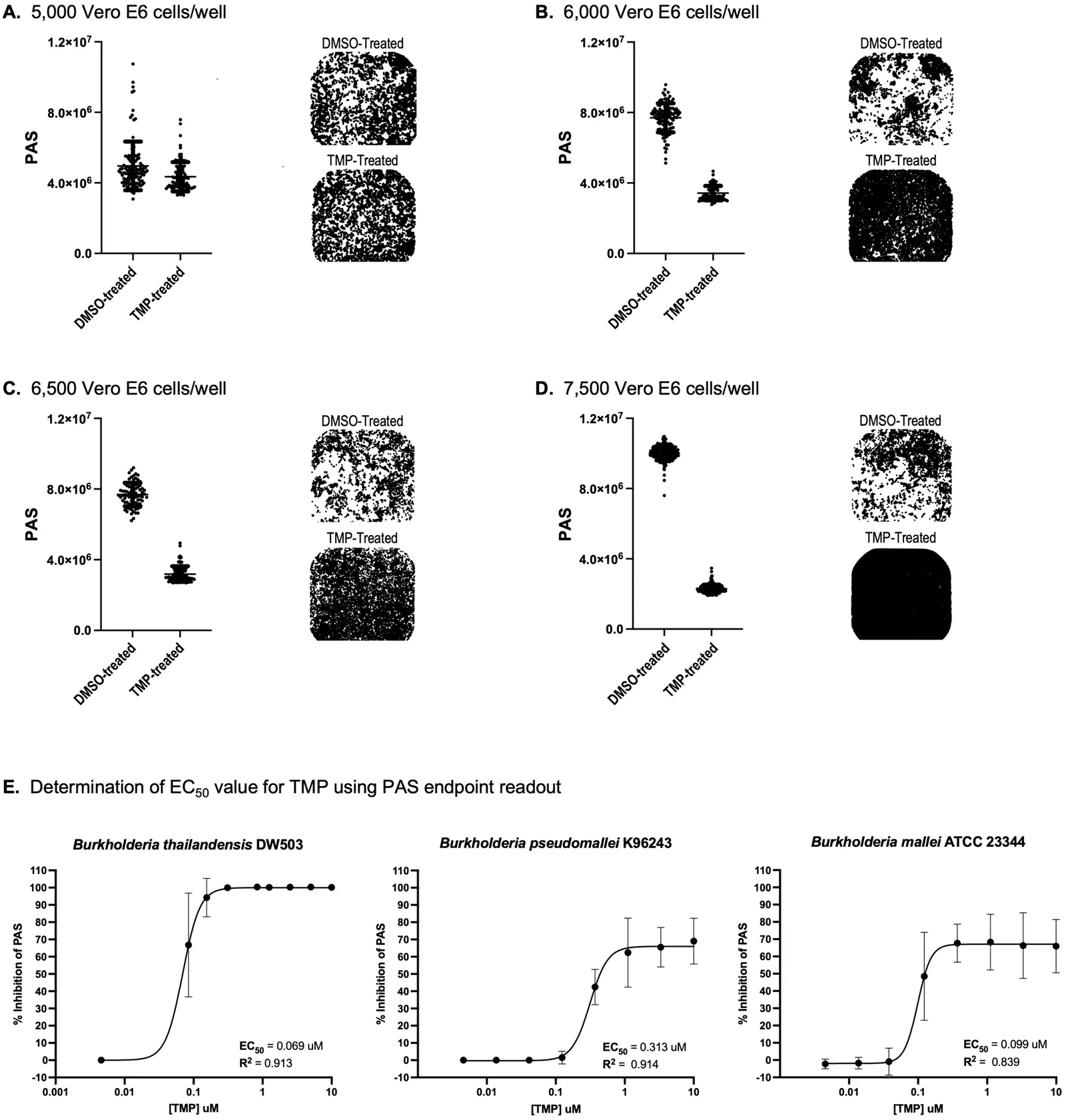
Optimization of 384-well infection assay with benchmark drug blocking intracellular replication of *Burkholderia thailandensis* strain DW503. Panels A-D: Monolayers of Vero E6 cells were infected with 10 µL of bacterial suspension containing 10^5^ CFU, incubated 3 hours at 37^o^C to allow bacterial attachment and entry, overlayed with medium supplemented with 100 ug/mL kanamycin (to kill extracellular bacteria) and 10 µM trimethoprim (to block intracellular replication of bacteria, TMP-treated), incubated at 37^o^C for 48 hours to allow plaque formation, treated with fixative containing Hoechst dye, imaged with an ImageXpress Micro Confocal High Content Imaging System, and analyzed with the MetaXpress software and our series of filters and masks; DMSO was used in place of TMP in control wells. Representative images are shown. Plaque Area Summed (PAS) read out values are expressed in µm^2^ and n = 192 replicate wells were used for each condition (DMSO-treated, TMP-treated). **Panel E**: Graphs represent sigmoidal dose response curves to determine the concentrations of TMP that inhibit the intracellular replication of *Bt*, *Bp* and *Bm* by 50% (based on the reduction in PAS readout values). EC_50_ values were calculated using the GraphPad Prism software by plotting percent inhibition of PAS (TMP-treated vs DMSO-treated) as a function of TMP concentration (n = 6 independent replicate experiments).

Prior to screening libraries of compounds, the assay was calibrated with the benchmark protection drug trimethoprim (TMP) in a 12-point dose response experiment with concentrations ranging from 847 pM to 50 µM. The antibiotic was chosen based on published data demonstrating its ability to block *Burkholderia* intracellular replication and plaque formation in cell culture assays [22]. Different seeding densities of Vero E6 cells (5-, 6-, 6.5- and 7.5x10^3^ cells per well) were also assessed to optimize PAS values. Fig 3A-3D shows the results of these experiments. Briefly, monolayers were infected with 10^5^ CFU of *Bt* DW503 in 10 µL, incubated for 3 hours to allow bacterial attachment and entry into cells, overlayed with medium containing 100 µg/mL kanamycin (to kill extracellular bacteria) and 10 µM TMP (to block *Burkholderia* intracellular replication), incubated for 48 hours to allow plaque formation, fixed and stained with Hoechst dye, imaged, and analyzed with the MetaXpress software and our series of filters and masks; DMSO was used in lieu of TMP in control wells. We found that a seeding density of 7.5x10^3^ cells/well (Fig 3D) yielded the best results and the greatest resolution of PAS between permissive (DMSO-treated) and non-permissive (TMP-treated) conditions, presumably by forming a more compact and uniform monolayer (compare TMP-treated image of Fig 3D to that of Fig 3A). Fig 3E summarizes the results of dose response curve experiments using the optimal host cell seeding density of 7.5x10^3^ cells/well.

**Fig 4:**
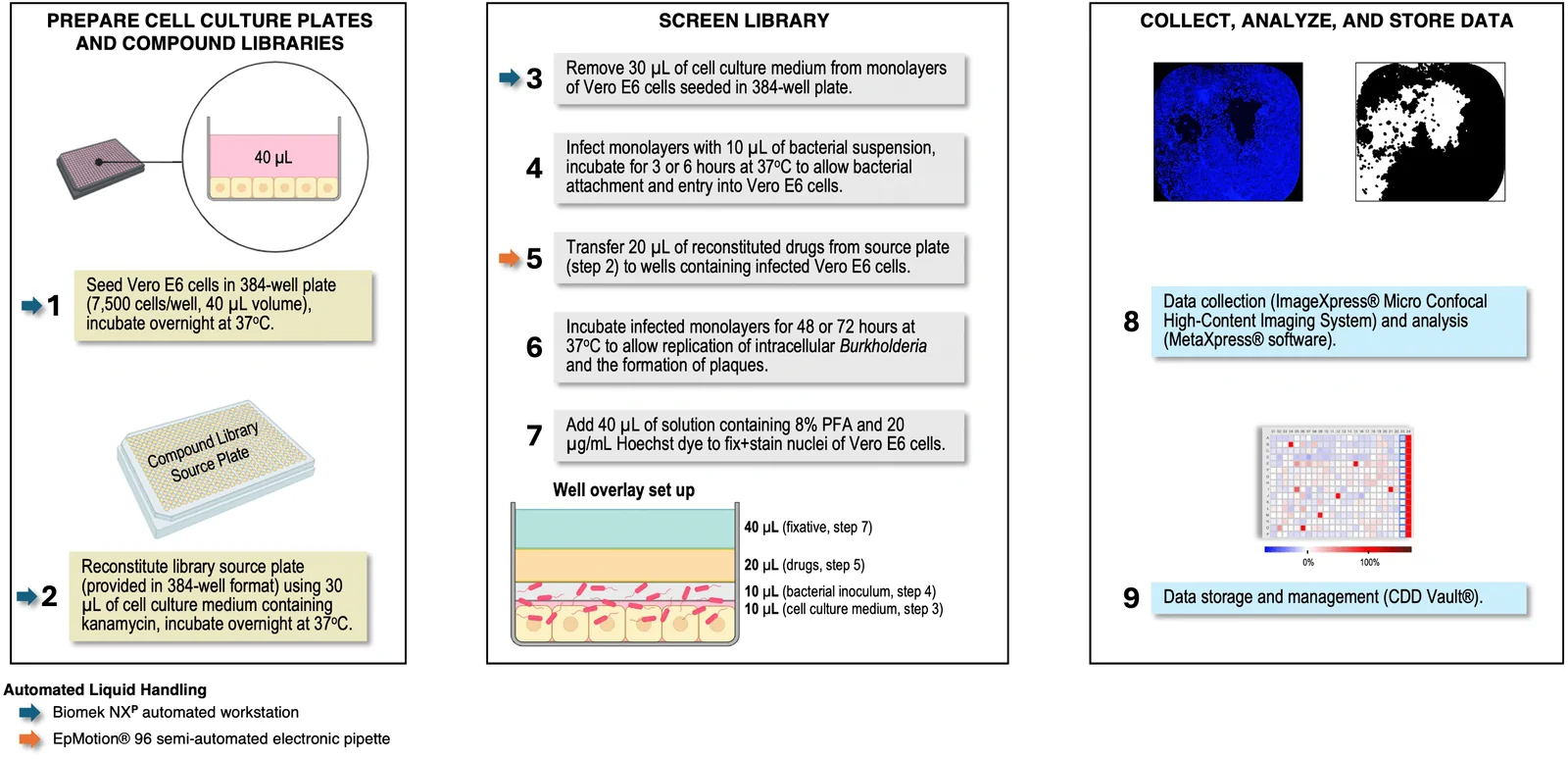
Workflow of high-content imaging, high-throughput screening platform for the discovery of compounds that protect against *Burkholderia* intracellular replication. Images were created with BioRender.com.

**Fig 5:**
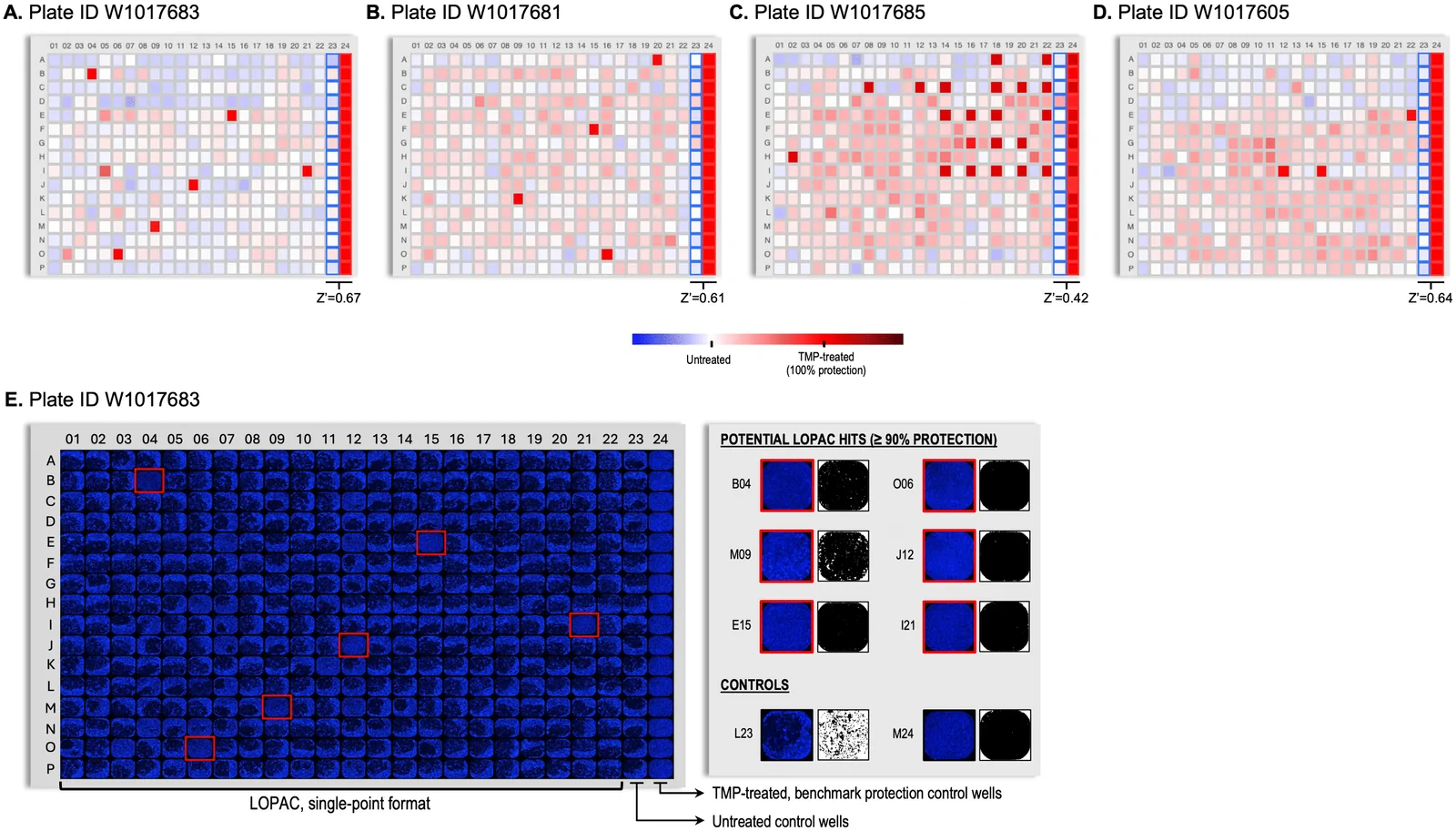
LOPAC screen to identify compounds blocking intracellular replication of *Burkholderia thailandensis* strain DW503. Monolayers of Vero E6 cells were infected with 10 µL of bacterial suspension containing 10^5^ CFU, incubated 3 hours at 37^o^C to allow bacterial attachment and entry, overlayed with medium supplemented with 100 µg/mL kanamycin (to kill extracellular bacteria) and 10 µM of individual LOPAC small molecules (columns 1-22) or TMP (column 24), incubated at 37^o^C for 48 hours to allow plaque formation, treated with fixative containing Hoechst dye, imaged with an ImageXpress Micro Confocal High Content Imaging System, and analyzed with the MetaXpress software and our series of filters and masks. Panels A-D show heat maps generated using the CDD Vault management system and representing percent protection against *Bt* intracellular replication relative to the benchmark drug TMP. The Z’-factor comparing untreated (column 23) and TMP-treated (column 24) wells is shown underneath each heat map. Panel E shows representative high-content imaging data used to generate the heat map in panel A. Red squares highlights LOPAC compounds providing ≥ 90% protection against intracellular bacterial replication.

Following optimization, the infection protocol and analysis module for high-content imaging data were tested on a small-scale to discover drugs that protect against bacterial intracellular replication and plaque formation. This was accomplished using a Library of Pharmacologically Active Compounds (LOPAC) that contains 1,280 small molecules covering major classes of druggable targets known for their availability and safety in humans. Three copies of the LOPAC were obtained from Calibr-Skaggs (Scripps Research) in blinded single-point format and the workflow for screening the library is outlined in Fig 4. Each copy consisted of 4 assay plates, and the LOPAC was tested on 3 separate occasions for compounds active against *Bt* DW503 at a concentration of 10 µM. Fig 5 shows the results of testing one copy of the library. All data points were fed into CDD Vault and analyzed to produce heat maps representing the activity of individual compounds relative to the TMP benchmark. As depicted in Fig 5, each assay plate included 16 replicate wells treated with TMP in column 24. From these, we calculated an average PAS that was used as the reference point for 100% protection against intracellular replication. Each assay plate also included 16 untreated control wells in column 23, which were used to verify the reproducibility of infection and plaque formation.

Using this approach, we identified 15 compounds providing ≥ 90% protection in at least 2 of the 3 independent biological replicates. The blinded hits were resupplied in 8-point dose response format and tested as described above for the LOPAC. Compounds showing both a sigmoidal dose response curve and EC_50_ value < 9.99 µM were considered successful hits and unblinded. Of note, EC_50_ values reported herein reflect inhibition of intracellular bacterial replication and plaque formation rather than direct cytotoxicity of the compounds to host cells.

As shown in Table 1, the 15 compounds had EC_50_ values ranging from 80 nM to 9.66 µM and were disclosed. Twelve compounds were antibiotics and the benchmark protection drug TMP was identified further validating the screen. The other 3 successful hits were likely host-directed agents based on annotation. Taken together, these results demonstrate our ability to establish a high-content imaging, high-throughput screening platform for the discovery of drugs that can disrupt *Burkholderia* intracellular replication and plaque formation. We refer to this entire process as **S**creen for **C**ompounds blocking **R**eplication of **I**ntracellular ***B****urkhold****E****ria* (SCRIBE).

**Table 1:** EC_50_ values of LOPAC hits protecting against intracellular replication of *Burkholderia thailandensis* DW503.

Compound type	Compound name	Mechanism of Action	^a^EC_50_	^b^R^2^
Antibiotics interfering with DNA synthesis	Trimethoprim (TMP)	Inhibits dihydropholate reductase (DHFR)	0.09	0.78
	Sulfaphenazole	Sulfonamide-class; inhibits dihydropteroate synthase (DHPS)	1.14	0.99
Antibiotics interfering with protein synthesis	Doxycycline	Tetracycline-class; binds 30S subunit of bacterial ribosome and hinders binding of tRNA	0.10	1.00
	Demeclocycline	Tetracycline-class; binds 30S subunit of bacterial ribosome and hinders binding of tRNA	0.19	0.99
	Minocycline	Tetracycline-class; binds 30S subunit of bacterial ribosome and hinders binding of tRNA	2.13	0.99
	Sodium fusidate	Prevents the turnover of elongation factor G from the bacterial ribosome	6.11	0.95
Antibiotics interfering with DNA replication	Gemifloxacin	Fluoroquinolone-class; inhibits DNA gyrase	0.19	0.99
	Finafloxacin	Fluoroquinolone-class; inhibits DNA gyrase	0.66	0.98
	Lomefloxacin	Fluoroquinolone-class; inhibits DNA gyrase	0.21	0.99
	Ofloxacin	Fluoroquinolone-class; inhibits DNA gyrase	1.52	0.94
	Nalidixic acid	Fluoroquinolone-class; inhibits DNA gyrase	7.22	0.98
	Oxolinic acid	Quinolone-class; inhibits DNA gyrase	9.66	0.99
Host-directed compounds	Phorbol 12-myristate 13-acetate	Protein kinase C activator; stimulates T-cell activation and cytokine production	0.08	0.95
	Diphenyleneiodonium	Endothelial nitric oxide synthase inhibitor	0.58	0.99
	1,10-Phenanthroline monohydrate	Metalloprotease inhibitor; chelates iron, zinc and other divalent metals	2.96	0.98

^a^Compounds were resupplied in 8-point dose response format with concentrations ranging from 0.0845 to 9.66 µM and tested on 3 separate occasions; average EC_50_ values are shown in µM. Compounds with EC_50_ values ≥ 9.99 µM were considered unconfirmed and are not listed. ^b^Average R^2^ values are shown. R² values indicate the goodness-of-fit of the sigmoidal dose-response curves to the four-parameter logistic model.

**Fig 6:**
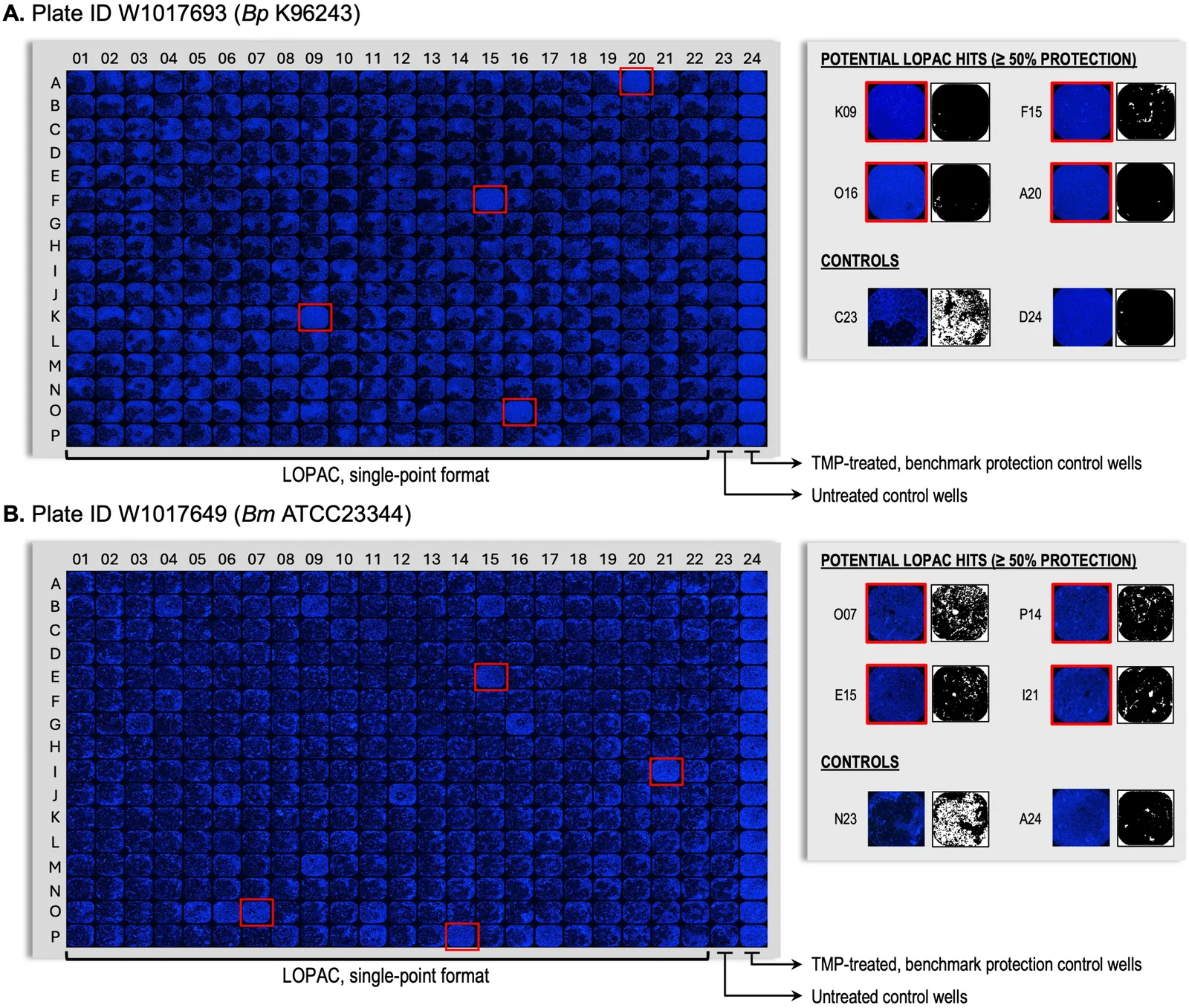
LOPAC screen to identify compounds blocking intracellular replication of the wild-type strains *Burkholderia pseudomallei* K96243 and *Burkholderia mallei* ATCC 23344. Monolayers of Vero E6 cells (7,500/well) were infected with 10 µL of bacterial suspension, incubated at 37^o^C to allow bacterial attachment and entry, overlayed with medium supplemented with 1000 µg/mL kanamycin (to kill extracellular bacteria) and 10 µM of individual LOPAC small molecules (columns 1-22) or TMP (column 24), incubated at 37^o^C to allow plaque formation, treated with fixative containing Hoechst dye, imaged with an ImageXpress Micro Confocal High Content Imaging System, and analyzed with the MetaXpress software and our series of filters and masks. Panel A shows a representative plate from the single-point *Bp* LOPAC screen, illustrating the visual distinction between hit and non-hit compounds following infection of monolayers with 10^4^ CFU of *Bp* K96243, incubated for 3 hours to allow bacterial attachment and entry, and incubated for 48 hours to allow intracellular replication and plaque formation. Panel B shows a representative plate from the single-point *Bm* LOPAC screen, illustrating the visual distinction between hit and non-hit compounds following infection of monolayers infected with 10^5^ CFU of *Bm* ATCC 23344, incubated for 6 hours to allow bacterial attachment and entry, and incubated for 72 hours to allow intracellular replication and plaque formation. Shown are representative high-content imaging data. Red squares highlight LOPAC compounds providing ≥ 50% protection against intracellular bacterial replication.

**Fig 7:**
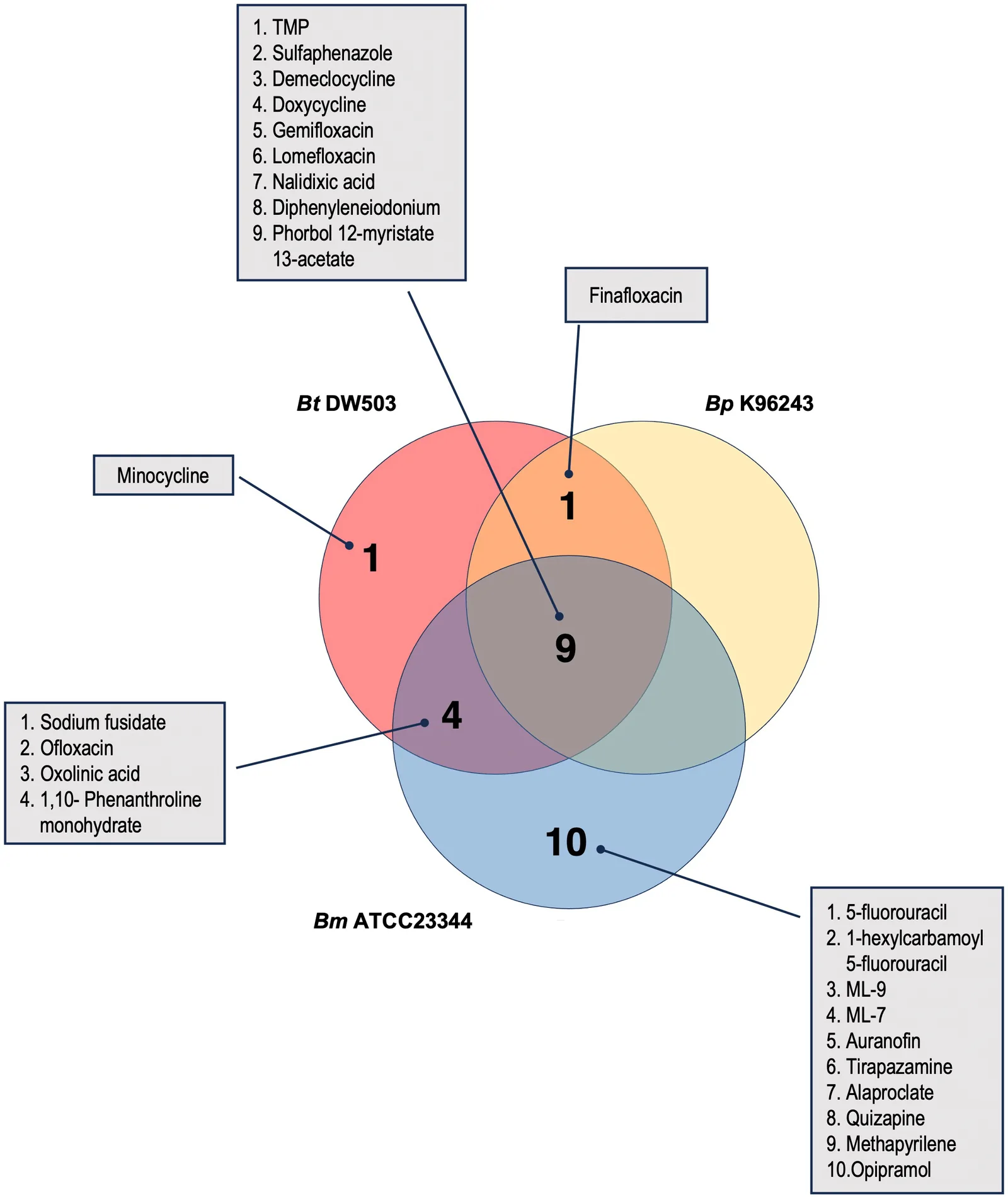
Comparison of LOPAC hits with activity against *Burkholderia thailandensis* DW503*, Burkholderia pseudomallei* K96243 and *Burkholderia mallei* ATCC 23344.

After validating the platform with *Bt* DW503, SCRIBE was used in the BSL-3 laboratory to identify LOPAC compounds active against the wild-type (WT) strains *Bp* K96243 and *Bm* ATCC 23344 with some modifications to the infection assay. For *Bp* K96243, monolayers were infected with fewer organisms (10^4^ CFU) and a higher kanamycin concentration of 1000 µg/mL to kill extracellular bacteria. The other parameters remained the same as in assays with *Bt* DW503 (seeding density of 7,500 Vero E6 cells/well, inoculum volume of 10 µL, 3-hr incubation at 37^o^C to allow bacterial attachment and entry into cells, intracellular replication and plaque formation period of 48 hours at 37^o^C). Under these conditions, the TMP benchmark protection drug showed an EC_50_ value of 0.313 µM and R^2^ coefficient of 0.914 against *Bp* K96243 in optimization experiments (Fig 3E). For infection with *Bm* ATCC 23344, the bacterial attachment and cell entry period was extended to 6 hours, the incubation time for intracellular replication and plaque formation was increased to 72 hours, and the higher kanamycin concentration of 1000 µg/mL was used in the overlay medium. Other variables (seeding density of Vero E6 cells, infection volume of 10 µL, inoculum of 10^5^ CFU) were kept the same as in assays with *Bt* DW503. Under these conditions, we calculated an EC_50_ for TMP of 0.099 µM with an R^2^ of 0.839 for *Bm* ATCC 23344 (Fig 3E). Fig 6 shows representative high-content imaging data of LOPAC screens with the WT *Bp* and *Bm* organisms in which the above modifications to the infection assay were implemented. In these experiments, compounds providing ≥ 50% protection against intracellular replication and plaque formation were considered potential hits and resupplied/tested in 8-point dose response assays. Table 2 (10 compounds, confirmation rate of 22.7%) and Table 3 (23 compounds, confirmation rate of 37.7%) list unblinded LOPAC drugs reproducibly showing a sigmoidal dose response curve as well as EC_50_ < 9.99 µM against *Bp* K96243 and *Bm* ATCC 23344, respectively. Comparison of the successful hits also revealed 9 compounds with bioactivity against all three *Burkholderia* species tested consisting of 7 antibiotics (including the protection benchmark TMP) and the host-directed drugs Phorbol 12-myristate 13-acetate (PMA) and diphenyleneiodonium (Fig 7). Ten compounds were found to have *Bm*-specific activity, 8 of which with previously identified eukaryotic cell targets. The tetracycline-class antibiotic minocycline was found to have bioactivity against *Bt*, exclusively. The LOPAC screen did not identify compounds with unique activity against *Bp*.

**Table 2:** EC_50_ values of LOPAC hits protecting against intracellular replication of *Burkholderia pseudomallei* K96243^a.^

Compound type	Compound name	Mechanism of Action	^b^EC_50_	^c^R^2^
Antibiotics interfering with DNA synthesis	Trimethoprim (TMP)	Inhibits dihydropholate reductase (DHFR)	0.32	0.81
	Sulfaphenazole	Sulfonamide-class; inhibits dihydropteroate synthase (DHPS)	7.12	0.81
Antibiotics interfering with protein synthesis	Doxycycline	Tetracycline-class; binds 30S subunit of bacterial ribosome and hinders binding of tRNA	0.45	0.99
	Demeclocycline	Tetracycline-class; binds 30S subunit of bacterial ribosome and hinders binding of tRNA	1.18	0.64
Antibiotics interfering with DNA replication	Gemifloxacin	Fluoroquinolone-class; inhibits DNA gyrase	1.04	0.94
	Lomefloxacin	Fluoroquinolone-class; inhibits DNA gyrase	1.37	1.00
	Finafloxacin	Fluoroquinolone-class; inhibits DNA gyrase	2.02	0.99
	Nalidixic acid	Quinolone-class; inhibits DNA gyrase	4.07	0.95
Host-directed compounds	Phorbol 12-myristate 13-acetate	Protein kinase C activator; stimulates T-cell activation and cytokine production	0.02	0.98
	Diphenyleneiodonium	Endothelial nitric oxide synthase inhibitor	1.56	0.99

^a^Compounds providing protection against intracellular replication in 2 independent rounds of LOPAC screening. ^b^Compounds were resupplied in 8-point dose response format with concentrations ranging from 0.00467 to 9.99 µM and tested on 2 separate occasions; average EC_50_ values are shown in µM. Compounds with EC_50_ values > 9.99 µM were considered unconfirmed and are not listed. ^c^Average R^2^ values are shown. R² values indicate the goodness-of-fit of the sigmoidal dose-response curves to the four-parameter logistic model.

**Table 3:** EC_50_ values of LOPAC hits protecting against intracellular replication of *Burkholderia mallei* ATCC 23344^a.^

Compound type	Compound name	Mechanism of Action	^b^EC_50_	^c^R^2^
Antibiotics interfering with DNA synthesis	Trimethoprim (TMP)	Inhibits dihydropholate reductase (DHFR)	0.07	0.65
	Sulfaphenazole	Sulfonamide-class; inhibits dihydropteroate synthase (DHPS)	0.67	0.98
	5-fluorouracil	Inhibits thymidylate synthase and the production of deoxythymidine monophosphate	4.07	0.78
	1-hexylcarbamoyl-5-fluorouracil	Inhibits thymidylate synthase and the production of deoxythymidine monophosphate	7.93	0.80
Antibiotics interfering with protein synthesis	Demeclocycline	Tetracycline-class; binds 30S subunit of bacterial ribosome and hinders binding of tRNA	0.05	0.98
	Doxycycline	Tetracycline-class; binds 30S subunit of bacterial ribosome and hinders binding of tRNA	0.03	0.99
	Sodium fusidate	Prevents the turnover of elongation factor G from the bacterial ribosome	7.38	0.78
Antibiotics interfering with DNA replication	Gemifloxacin	Fluoroquinolone-class; inhibits DNA gyrase	0.05	0.99
	Ofloxacin	Fluoroquinolone-class; inhibits DNA gyrase	0.33	0.99
	Lomefloxacin	Fluoroquinolone-class; inhibits DNA gyrase	0.08	0.99
	Oxolinic acid	Quinolone-class; inhibits DNA gyrase	5.82	0.69
	Nalidixic acid	Quinolone-class; inhibits DNA gyrase	2.17	0.97
Host-directed compounds	Diphenyleneiodonium	Endothelial nitric oxide synthase inhibitor	0.55	0.99
	ML-9	Myosin light chain kinase inhibitor	1.51	0.92
	ML-7	Myosin light chain kinase inhibitor	1.91	0.96
	1,10-Phenanthroline monohydrate	Metalloprotease inhibitor; chelates iron, zinc and other divalent metals	1.99	0.99
	Auranofin	Antirheumatic drug	2.22	0.69
	Tirapazamine	Cytotoxic for hypoxic cells in tumors, induces DNA damage	3.35	0.97
	Phorbol 12-myristate 13-acetate	Protein kinase C activator; stimulates T-cell activation and cytokine production	3.39	0.66
	Alaproclate	Serotonin reuptake inhibitor	3.41	0.97
	Quipazine	Serotonin reuptake inhibitor	5.09	0.95
	Methapyrilene	H1-receptor antagonist; antihistamine and anticholinergic agent	7.82	0.54
	Opipramol	Tricyclic antidepressant; sigma 1 and 2 receptor agonist, moderate antagonist of dopamine D2, serotonin 5HT2, and histamine H1 receptors	9.48	0.883

^a^Compounds providing protection against intracellular replication in 2 independent rounds of LOPAC screening. ^b^Compounds were resupplied in 8-point dose response format with concentrations ranging from 0.00467 to 9.99 µM and tested on 3 separate occasions; average EC_50_ values are shown in µM. Compounds with EC_50_ values > 9.99 µM were considered unconfirmed and are not listed. ^c^Average R^2^ values are shown. R² values indicate the goodness-of-fit of the sigmoidal dose-response curves to the four-parameter logistic model.

### SCRIBE reproducibly identifies drugs with bioactivity against *Bt*, *Bp*, and *Bm* from a large library of compounds

At the time of our screens, the **Re**purposing, **F**ocused **R**escue, and **A**ccelerated **ME**dchem library (ReFRAME) contained 13,558 curated compounds that have undergone clinical development or thorough preclinical profiling [24], making the library a high value resource for the discovery of therapies against emerging infectious diseases. With this in mind, we obtained copies of the ReFRAME from Calibr-Skaggs in blinded single-point format and screened the library following the workflow of Fig 4 for compounds with bioactivity against *Bt* DW503, *Bp* K96243, and *Bm* ATCC 23344. As described above for LOPAC and illustrated in Fig 5, each ReFRAME assay plate included 16 untreated control wells in column 23 and 16 wells treated with the benchmark protection drug TMP in column 24. The library was first tested outside of the BSL-3 laboratory using *Bt* DW503 and comparison of benchmark and untreated control wells across all assay plates indicates highly reproducible PAS values with an average Z’-factor of 0.72 (S1 Table). TMP-treated wells showed an average PAS of 4.56x10^4^ µm^2^ and > 99.9% protection against intracellular replication while the average PAS readout value for untreated wells was 6.74x10^6^ µm^2^ (S1 Table). The screen identified 205 ReFRAME compounds that reproducibly provide ≥ 90% protection at a concentration of 10 µM. The blinded hits were resupplied in 8-point dose response format and tested as 2 biological replicates. Compounds showing both sigmoidal dose response curve and EC_50_ value < 9.99 µM were considered successful hits and unblinded. As shown in S2 Table, 161 compounds with EC_50_ values ranging from 30 nM to 9.97 µM were disclosed (confirmation rate of 78.5%). Most bioactive drugs belong to major classes of antibiotics (128/161, 79.5%), namely 12 inhibitors of dihydropholate reductase (DHFR), 25 sulfonamides, 11 tetracyclines, 69 fluoroquinolones, and 11 quinolones. SCRIBE also identified 10 host-directed compounds, four of which derivatives of the DNA topoisomerase I inhibitor and anti-cancer drug Camptothecin.

**Fig 8:**
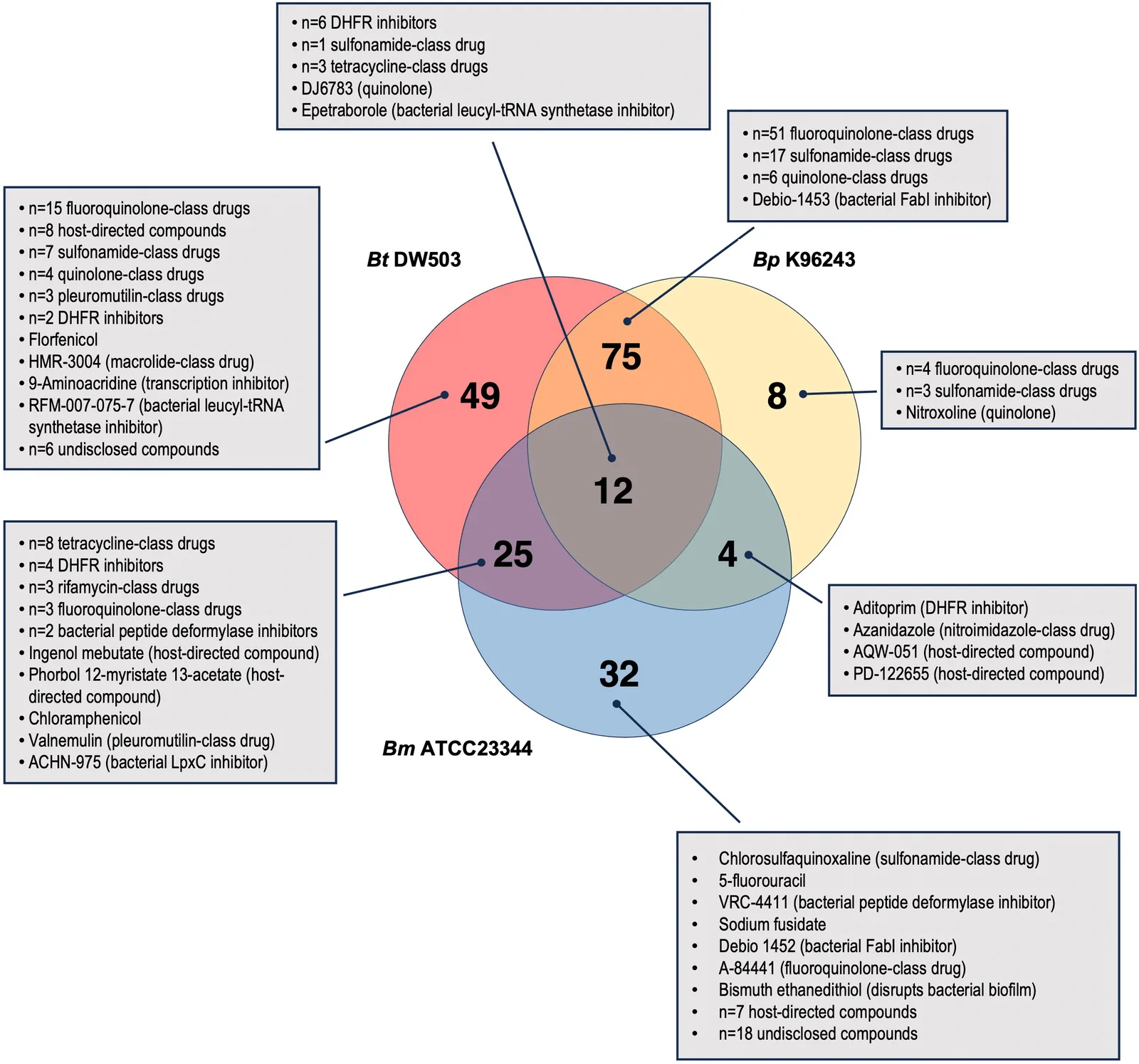
Comparison of ReFRAME hits with activity against *Burkholderia thailandensis* DW503*, Burkholderia pseudomallei* K96243 and *Burkholderia mallei* ATCC 23344.

The library was next tested in the BSL-3 laboratory with *Bp* K96243. The comparative analysis of PAS values is shown in S3 Table and indicates reproducibility for the untreated and TMP-treated control wells across all assay plates. SCRIBE identified 402 compounds that provide ≥ 70% protection against intracellular replication of *Bp* K96243 at a concentration of 10 µM. To prioritize the number of leads to test in dose response experiments, the 402 compounds were resupplied by Calibr-Skaggs in a modified single-point format where each drug was provided at a concentration of 2 µM in triplicate wells (ReFRAME 3x SP). Using a ≥ 75% protection threshold, we determined that 108 of the 402 compounds were still bioactive at the lower testing concentration. These compounds were resupplied/tested in 8-point dose response assays and 99 showed sigmoidal dose response curves as well as EC_50_ values ranging from 20 nM to 9.99 µM (S4 Table, confirmation rate of 91.7%). Similar to the outcome of the *Bt* DW503 ReFRAME screen, most drugs interfering with *Bp* K96243 intracellular replication (94/99, 95%) belong to major classes of antibiotics including inhibitors of 7 DHFR, 21 sulfonamides, 3 tetracyclines, 55 fluoroquinolones, and 8 quinolones. The *Bp* screen also identified the host-directed compounds AQW-051 and PD-122655, both of which target receptors on the surface of mammalian cells [25]. Side-by-side comparison of successful hits obtained in the *Bt* DW503 and *Bp* K96243 ReFRAME screens shows considerable overlap. As illustrated in Fig 8, we found that 87 leads with bioactivity against *Bp* K96243 also protect against intracellular replication of *Bt* DW503. The majority of the compounds with overlapping activity were sulfonamides (*n* = 18) and fluoroquinolones (*n* = 51).

The ReFRAME was also tested with *Bm* ATCC 23344 in the BSL-3 laboratory. Comparison of benchmark and untreated control wells across all assay plates indicates reproducible PAS readouts; TMP-treated wells showed an average PAS of 1.90x10^6^ µm^2^ and 99.78% protection against intracellular replication while the PAS for untreated wells was 8.96x10^6^ µm^2^ (S5 Table). Given the considerable number of sulfonamides and fluoroquinolones identified in the ReFRAME screens with *Bt* DW503 and *Bp* K96243, we requested that Calibr-Skaggs exclude these two classes of antibiotics from further reconfirmation. The *Bm* screen identified 308 compounds (after exclusion of sulfonamides and fluoroquinolones) providing ≥ 50% protection at a concentration of 10 µM. To prioritize the number of compounds to test in dose response experiments, the 308 *Bm* hits were resupplied at a concentration of 2 µM in the ReFRAME 3x SP format outlined above for the *Bp* K96243 screen. Using a ≥ 75% protection benchmark, we determined that 73 of these compounds were still bioactive at the lower testing concentration. These compounds were resupplied/tested in 8-point dose response assays and all (confirmation rate of 100%) showed sigmoidal dose response curves and EC_50_ values ranging from 0.01 to 9.95 µM (S6 Table). Many bioactive drugs belong to classes of antibiotics including 11 inhibitors of DHFR, 11 tetracyclines, and 3 rifamycins. SCRIBE also identified 11 host-directed compounds including AQW-051 and PD-122655, which were discovered in the ReFRAME screen with *Bp* K96243. Eighteen compounds were not disclosed by Calibr-Skaggs due to proprietary restrictions arising from the material transfer and user agreements between our institution and Calibr-Skaggs, as well as upstream agreements with contributing pharmaceutical companies.

Comparison of the successful hits reveals 12 drugs with bioactivity against all three organisms including 6 inhibitors of DHFR, 3 tetracyclines, and the antibiotic epetraborole, which is a boron-heterocyclic compound that inhibits the bacterial enzyme leucyl-tRNA synthetase LeuRS. Epetraborole was recently shown to inhibit growth of *Bp in vitro* [26,27] and represents a novel class of antimicrobials in the *Burkholderia* field. Based on the results of the LOPAC and ReFRAME screens, we selected the following compounds with broad anti-*Burkholderia* activity for *in vivo* therapeutic efficacy testing: epetraborole (Fig 8; S2, S4 and S6 Tables), the host-directed compound diphenyleneiodonium (Tables 1–3, Fig 7), and the fluoroquinolone antibiotic levofloxacin (S2 and S4 Tables). The latter was selected on the basis of published data demonstrating *in vivo* protective efficacy against *Bp* and *Bm* infection and served as internal benchmark protection control drug [28,29].

### Compounds identified with SCRIBE protect mice against lethal aerosol infection with *Bp* and *Bm*

**Fig 9:**
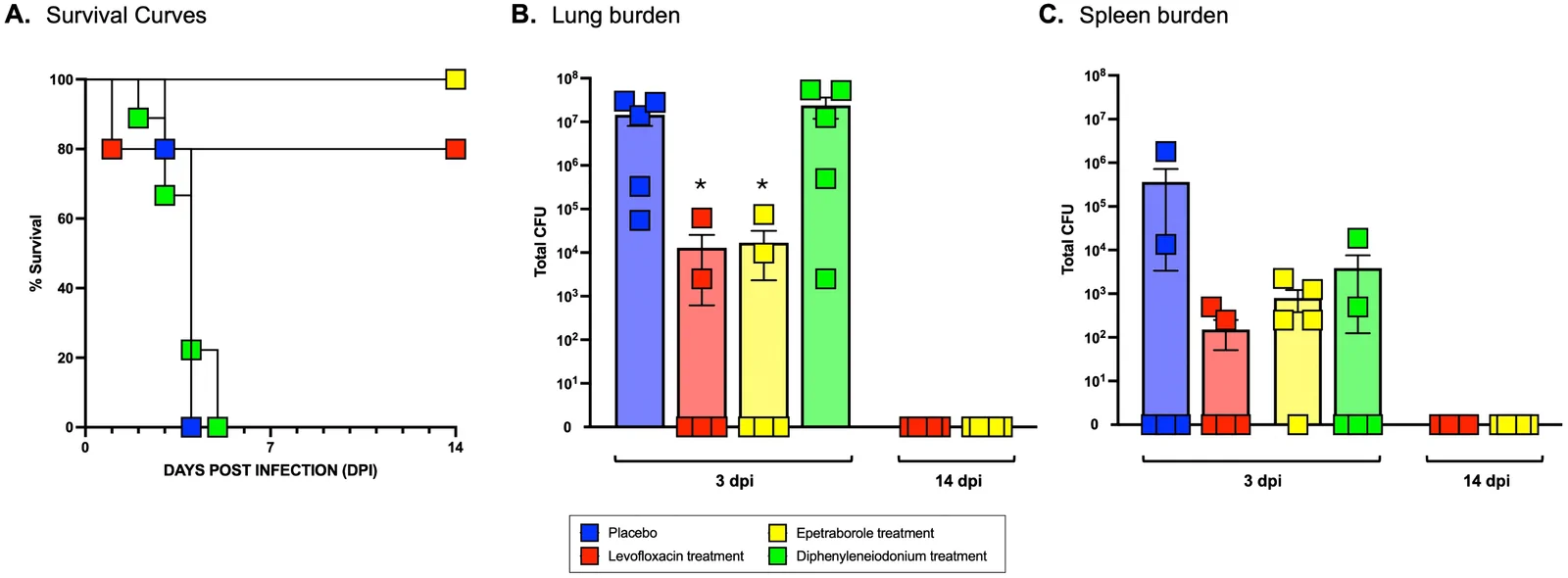
Treatment of mice infected with *Burkholderia pseudomallei* strain K96243 using bioactive compounds identified with SCRIBE. Female BALB/c mice (n = 15/experimental groups) were infected intratracheally using a Microsprayer to aerosolize 6 LD_50_ of wild-type *Bp* strain *Bp* K96243 (350 CFU) into the lungs. Animals were then monitored daily for signs of illness and morbidity for a period of 14 days. Treatment was administered daily via intraperitoneal injection starting on day 1 post-infection. The dosage for each injection: 5 mg/kg in 100 µL for levofloxacin, 20 mg/kg in 250 µL for epetraborole, 1 mg/kg in 250 µL for diphenyleneiodonium, and 250 µL of buffer used to formulate compound for placebo control animals. **Panel A**: Survival data. **Panels B and C**: Tissues were collected from animals, homogenized, diluted, and spread on agar plates to determine bacterial loads. Symbols represent individual animals; bars show mean total CFU ± SEM for each group. The asterisk indicates that the reduction in bacterial burden, compared to the placebo group, was statistically significant using an unpaired non-parametric Mann-Whitney *t* test (*P* < 0.05).

The *in vivo* therapeutic efficacy of levofloxacin, epetraborole and diphenyleneiodonium was evaluated using a mouse model of aerosol infection developed by our laboratory wherein animals are inoculated intratracheally with a Microsprayer nebulizing device to deliver bacteria into the lungs [30]. BALB/c mice were challenged with the equivalent of 6 LD_50_ of *Bp* K96243 and treatment was administered once daily via intraperitoneal injection, with the first dose given 24 hours post-challenge. Mice in the placebo control group were injected with the buffer used to formulate compounds. As shown in Fig 9A, all animals in the placebo and diphenyleneiodonium treatment groups reached humane endpoints by day 5 post-infection. In contrast, 100% of mice treated with epetraborole and 80% of animals given levofloxacin survived for the duration of the study. The bacterial burden in the lungs (Fig 9B) and spleen (Fig 9C) of survivors was also determined on days 3 and 14 post-challenge. Mice given epetraborole and levofloxacin showed a statistically significant reduction in the number of organisms colonizing the lungs on day 3 post-infection but not the spleen, compared to mice in the placebo group. On day 14, no bacteria could be detected in the tissues of 100% (5/5) of animals treated with epetraborole and 100% (4/4) of those given levofloxacin.

**Fig 10:**
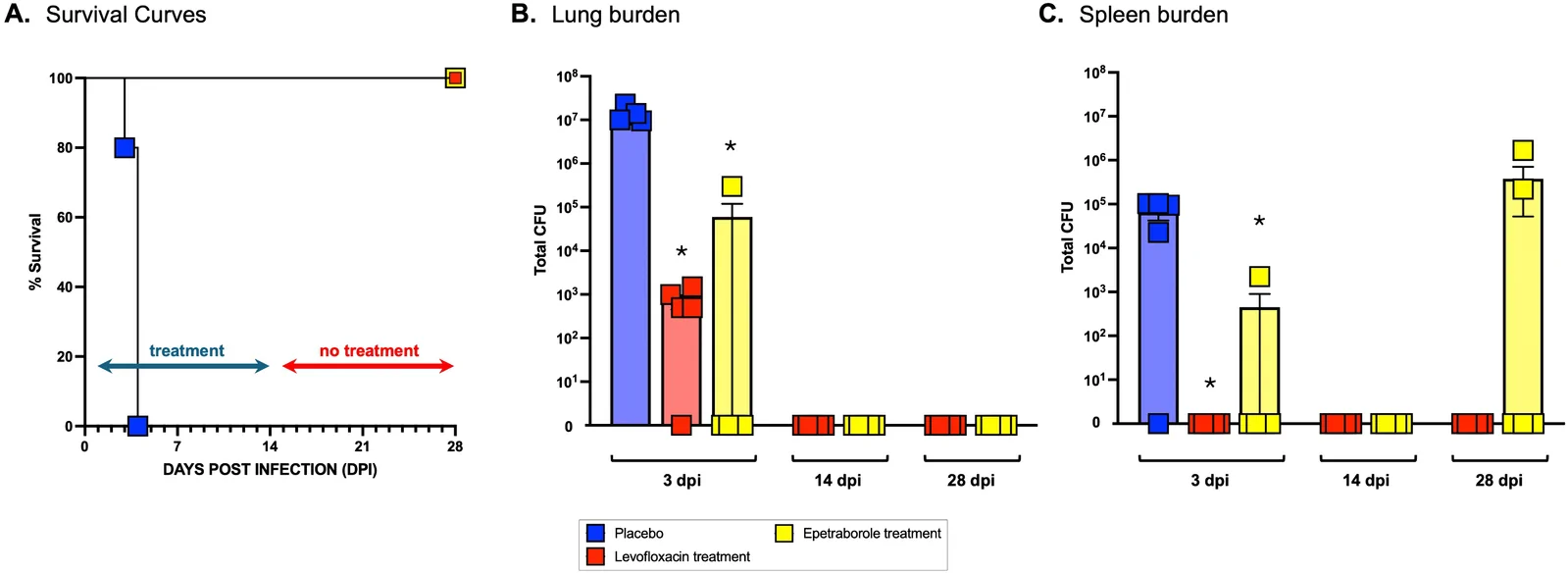
Treatment of mice infected with *Burkholderia pseudomallei* strain K96243 using levofloxacin or epetraborole. Female BALB/c mice (n = 15/experimental groups) were infected intratracheally using a Microsprayer to aerosolize 7 LD_50_ of wild-type *Bp* strain *Bp* K96243 (430 CFU) into the lungs. Animals were then monitored daily for signs of illness and morbidity for a period of 28 days. Treatment was administered daily via intraperitoneal injection starting on day 1 post-infection until day 14. The dosage for each injection: 5 mg/kg in 100 µL for levofloxacin, 20 mg/kg in 250 µL for epetraborole, and 250 µL of buffer used to formulate compounds for placebo control animals. **Panel A**: Survival data. **Panels B and C**: Tissues were collected from animals, homogenized, diluted, and spread on agar plates to determine bacterial loads. Symbols represent individual animals; bars show mean total CFU ± SEM for each group. The asterisk indicates that the reduction in bacterial burden, compared to the placebo group, was statistically significant using an unpaired non-parametric Mann-Whitney *t* test (*P* < 0.05).

To determine whether treating with epetraborole and levofloxacin for 14 days completely clears the infection, mice were challenged with 7 LD_50_ of *Bp* K96243 and monitored for a period of 28 days. The animals were given placebo, epetraborole and levofloxacin daily the first 14 days post-infection but did not receive treatment during the last 14 days of the experiment. All control animals injected with placebo succumbed to infection within 4 days post-challenge whereas 100% of mice in the epetraborole and levofloxacin treatment groups survived the entire length of the study (Fig 10A). On day 3 post-challenge, the animals treated with epetraborole and levofloxacin showed statistically significant reduction in the bacterial burden in both the lungs (Fig 10B) and spleen (Fig 10C) compared to the placebo group. Consistent with the data from the previous experiment, no bacteria could be detected in the lungs (Fig 10B) or spleen (Fig 10C) of survivors on day 14 post-challenge. On day 28, 5/5 mice (100%) treated with levofloxacin had no detectable organisms in tissues and 3/5 animals (60%) given epetraborole completely cleared bacteria from both spleen and lungs. Although the 28-day observation period demonstrates strong protective efficacy during the acute phase of infection, longer-term studies will be important to fully evaluate the ability of these compounds to prevent chronic infection, latency, and relapse, which are hallmark features of melioidosis and glanders.

**Fig 11:**
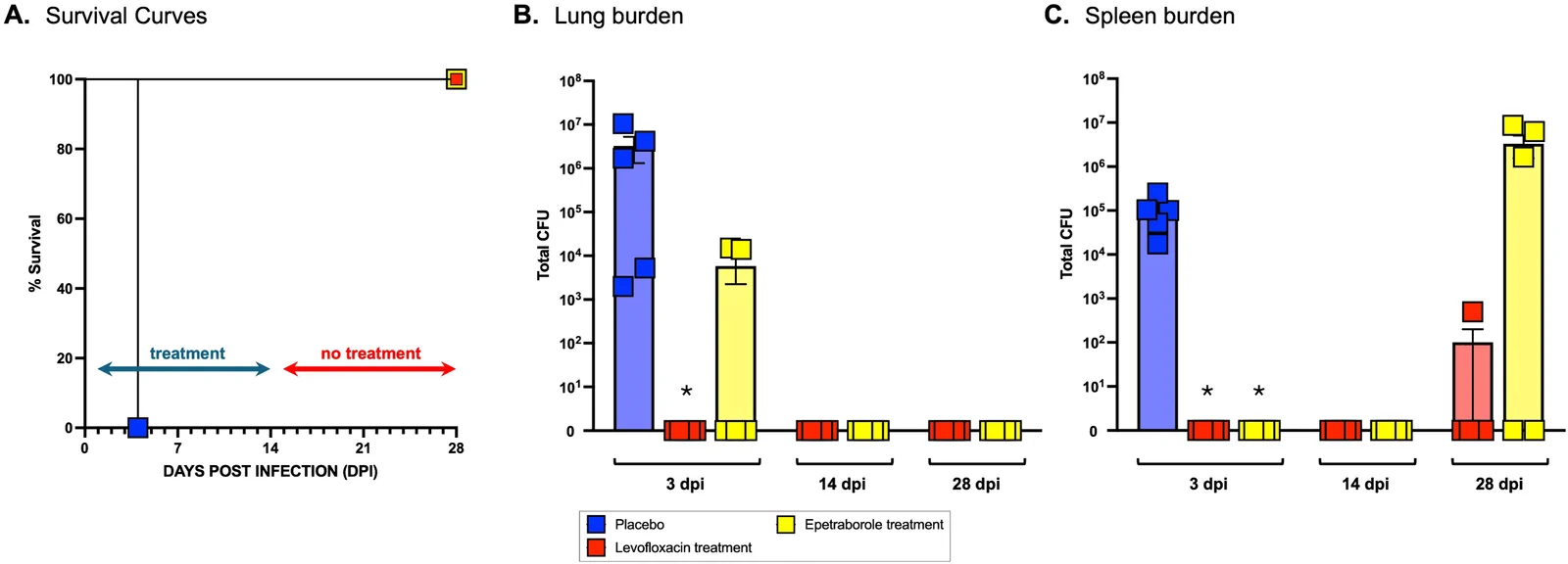
Treatment of mice infected with *Burkholderia mallei* strain ATCC 23344 using levofloxacin and epetraborole. Female BALB/c mice (n = 15/experimental groups) were infected intratracheally using a Microsprayer to aerosolize 4.5 LD_50_ of wild-type strain *Bm* ATCC 23344 (3,600 CFU) into the lungs. Animals were then monitored daily for signs of illness and morbidity for a period of 28 days. Treatment was administered daily via intraperitoneal injection starting on day 1 post-infection until day 14. The dosage for each injection: 5 mg/kg in 100 µL for levofloxacin, 20 mg/kg in 250 µL for epetraborole, and 250 µL of buffer used to formulate compounds for placebo control animals. **Panel A**: Survival data. **Panels B and C:** Tissues were collected from animals, homogenized, diluted, and spread on agar plates to determine bacterial loads. Symbols represent individual animals; bars show mean total CFU ± SEM for each group. The asterisk indicates that the reduction in bacterial burden, compared to the placebo group, was statistically significant using an unpaired non-parametric Mann-Whitney *t* test (*P* < 0.05).

To evaluate the breadth of protection by epetraborole and levofloxacin, mice were challenged with 4.5 LD_50_ of *Bm* ATCC 23344 and observed for signs of illness and morbidity for 28 days. As described above, the animals were treated daily starting on day 1 post-infection until day 14 but were not given treatment during the last 14 days of the study. As shown in Fig 11A, all mice in the placebo group reached humane endpoints by day 4 post-challenge whereas 100% of those treated with epetraborole and levofloxacin survived for the duration of the experiment. By the last day of treatment, no bacteria could be detected in the lungs or spleen of survivors (see 14 dpi in Fig 11B and Fig 11C, respectively). On day 28, 4/5 mice (80%) treated with levofloxacin had no detectable organisms in tissues and 2/5 animals (40%) given epetraborole completely cleared bacteria from both spleen and lungs. Taken together, the data demonstrate that epetraborole and levofloxacin provide excellent protection against lethal aerosol challenge with *Bp* and *Bm* and promote clearance of infection from major target organs.

## Discussion

The goal of this study was to establish SCRIBE, a high-throughput, cell-based screening platform capable of identifying small molecules that disrupt the intracellular replication of *Bp* and *Bm -* a key virulence trait that drives bacterial persistence in host tissues and contributes to the pathogenesis of melioidosis and glanders. Using high-content imaging coupled with a phenotypically anchored readout, SCRIBE enabled the systematic evaluation of nearly 14,800 repurposed, pharmacologically active compounds and identified 170 unique structures with bioactivity against *Bp* and/or *Bm*.

A key design feature of SCRIBE was the strategic use of the non-pathogenic strain *Bt* DW503 as a genetically relevant BSL-2 surrogate. The organism recapitulates hallmark intracellular behaviors of *Bp* and *Bm* in cell culture models including invasion, replication, intercellular spread, cell death, and subsequent plaque formation. These allowed for the optimization of inoculum, monolayer density, antibiotic overlay, incubation times, and image-analysis parameters without the operational constraints of Tier 1 Select Agent work at BSL-3. Although experiments with *Bp* and *Bm* required assay modifications to maintain reproducibility and sensitivity, SCRIBE’s modular workflow easily translated to BSL-3 conditions, supporting standardized cross-species screening.

Assay performance was validated using the benchmark protection antibiotic trimethoprim (TMP), a DHFR inhibitor that blocks bacterial folic acid synthesis. Treating infected cells with TMP reproducibly disrupted intracellular replication, cell death, and plaque formation across *Burkholderia* species. Preserved monolayer integrity seen with TMP treatment was a marked contrast to the extensive cytopathic effects observed in mock-treated controls. These phenotypes were quantified using the Plaque-Area-Summed (PAS) metric, which integrates fluorescence intensity thresholding with morphological masking of high-content images to distinguish intact monolayers from plaque- and syncytia-induced void areas. PAS provided a robust and reproducible endpoint readout across assay plates and supported precise EC_50_ determination for active compounds. As previously reported by other HTS campaigns, a Z’-factor ≥ 0.5 is generally considered indicative of a robust and reproducible assay, while values > 0.7 are regarded as excellent. SCRIBE exhibited good to excellent Z’-factors across the majority of independent screens. Regarding the *Bp* K96243 ReFRAME screen, some plates displayed lower Z’-factors due to technical challenges (as noted in the footnotes of S3 Table). These were addressed by implementing a modified single-point reconfirmation at 2 µM in triplicate (ReFRAME 3x SP format) followed by stringent 8-point dose-response testing using a ≥ 75% protection threshold. This triage strategy effectively prioritized true hits, resulting in a high confirmation rate of 91.7% and reliable EC_50_ values (S4 Table). Collectively, these performance metrics, together with the repeated identification of benchmark compounds such as trimethoprim and the strong correlation with *in vivo* efficacy, demonstrate the reproducibility, adaptability, rigor, and scalability of SCRIBE for large-format drug screening.

It should be noted that the reported EC_50_ values represent inhibition of intracellular *Burkholderia* replication and subsequent plaque formation, not direct cytotoxicity of the compounds to host cells. Compounds in the LOPAC and ReFRAME libraries have undergone prior safety and toxicity profiling for repurposing purposes. In our phenotypic assay, monolayer protection served as a surrogate for effective limitation of intracellular replication; overt host cell toxicity would have been evident as monolayer disruption. While dedicated cytotoxicity counter-screens in uninfected cells and optimization of compound concentrations could further refine selectivity indices, the current approach was sufficient to identify candidates that translated successfully to *in vivo* efficacy testing. This is consistent with the design of repurposing libraries, where compounds have established safety profiles.

Previous high-throughput studies have evaluated compound activity against *Bt*, *Bp*, and *Bm*, but focused on different endpoints and/or experimental contexts. For instance, Ross and colleagues screened the 400-compound MMV Pathogen Box library against *Bp* in broth culture, using turbidity as endpoint measure to identify structures with bacteriostatic or bactericidal activity [31]. Barker *et al* used the cell-viability reagent PrestoBlue and screened 61,250 compounds against *Bt* for molecules that potentiate the bactericidal activity of ceftazidime [32]. Li and coworkers utilized HiTES (High-Throughput Elicitor Screening), a chemical-genetics strategy that inserts a *lacZ* reporter into silent biosynthetic gene clusters (BGCs), for luminescence-based detection of gene induction in broth culture using the β-galactosidase reagent β-Glo [33]. HiTES was applied to screen a collection of 770 FDA-approved drugs against a panel of *Bt* recombinant *lacZ* strains, revealing that sub-inhibitory concentrations of diverse drugs can induce expression of otherwise silent BGCs. Unlike these approaches, which primarily interrogate extracellular planktonic growth, SCRIBE captures the host cell context of bacterial intracellular replication and cell-to-cell spread.

High-content imaging platforms similar to SCRIBE have been successfully applied to other intracellular bacterial pathogens, including *Mycobacterium tuberculosis*, *Legionella pneumophila*, *Salmonella spp*., and *Chlamydia trachomatis*. These assays typically quantify intracellular bacterial burden (via fluorescent reporters or staining) or host cell viability, often in macrophage-like cells. SCRIBE differs in its use of Vero E6 epithelial cells, which are robust, readily maintained, and highly permissive to infection with minimal specialized culture requirements compared to primary cells or macrophage lines. Reliance on a label-free cytopathic endpoint (plaque/syncytia formation quantified by PAS) and a focus on facultative intracellular pathogens that cause overt monolayer destruction further distinguish the platform. Importantly, the label-free nature of the PAS readout makes SCRIBE particularly well-suited for stringent high-containment (BSL-3) environments, where genetic manipulation of select agents to express fluorescent reporters is often restricted or unavailable, and the use of antibodies for labeling may be limited by biosafety and logistical constraints. This phenotypic approach enables direct assessment of both direct-acting antimicrobials capable of intracellular penetration and host-directed compounds that limit cell-to-cell spread—features particularly relevant for *Burkholderia spp.* and other pathogens that rely on actin-based motility and multinucleated giant cell formation. By capturing these complex phenotypes in a high-throughput format compatible with BSL-3 conditions, SCRIBE complements existing platforms and provides a flexible framework adaptable to additional intracellular bacteria and even viruses that induce cytopathic effects.

In a more conceptually aligned study, Bulterys and colleagues implemented a cell-based platform to screen a library of more than 220,000 compounds against *Bt* [22]. Their platform relied on HEK293 human kidney cells engineered to express enhanced Green Fluorescent Protein (HEK293-eGFP), enabling high-content fluorescence imaging to quantify plaque-induced void areas and other cytopathic effects during infection. This large-scale effort identified 268 hits, of which 32 demonstrated activity against *Bp* and/or *Bm*. SCRIBE builds on and extends this work by integrating a unified, cross-species screening pipeline – using both the LOPAC and ReFRAME libraries – under harmonized assay conditions. This standardized framework enables direct comparative analyses of compound activity across *Bt, Bp,* and *Bm*, facilitating the systematic discovery of broad‑spectrum as well as organism‑specific inhibitors of intracellular replication.

SCRIBE also identified both convergent and previously unrecognized chemotypes with activity against *Burkholderia* species. Expected antibiotic classes – fluoroquinolones, DHFR inhibitors, sulfonamides, tetracyclines – were repeatedly identified across all 3 species, underscoring the biological relevance and rigorous screening process of the platform. Beyond these, SCRIBE revealed novel scaffolds that expand the chemical space for therapeutic development against melioidosis and glanders. Among the most notable findings was epetraborole, a boron-heterocyclic compound that targets the bacterial enzyme leucyl-tRNA synthetase (LeuRS) and halts protein synthesis [34]. Epetraborole exhibited potent *in vitro* activity against *Bt, Bp* and *Bm* (Fig 8) with EC_50_ values from 0.36 to 1.92 µM and protected mice against lethal aerosol challenge with *Bp* and *Bm* (Figs 10 and 11). A second boron-heterocyclic compound from the ReFRAME library, RFM-007-075-7, also disrupted intracellular replication of *Bt* DW503 *in vitro* (S2 Table). Our results are consistent with recently published reports demonstrating epetraborole’s *in vivo* efficacy against *Bp* [26,35] and reinforce the therapeutic potential of boron-heterocyclic compounds as possible next generation antimicrobials for *Bp* and *Bm*.

In addition to boron-based inhibitors, SCRIBE identified pleuromutilins - a class of protein synthesis inhibitors not previously linked to anti-*Burkholderia* activity - as hits in our screens. Pleuromutilins share a trycyclic core containing five-, six-, and eight- membered rings and incorporate a characteristic sulfanyl glycolic ester moiety [36]. Their antibacterial activity stems from binding the peptidyl transferase center in the bacterial 50S ribosomal subunit and preventing the correct positioning of tRNAs in the A and P sites, which in turn blocks peptide bond formation in growing peptides. Although pleuromutilins have traditionally been used to treat infections caused by multidrug-resistant gram-positive organisms, newer derivatives such as lefamulin have expanded activity against gram-negative bacteria and are being explored as countermeasures for high-priority organisms including ESKAPE pathogens [37–39]. Within the ReFRAME library the pleuromutilins BC-3205, SB-268091, and tiamulin were effective inhibitors of *Bt* intracellular replication (S2 Table), while valnemulin showed dual activity against *Bt* and *Bm* (S2 and S6 Tables, respectively). The established pharmacological profiles and clinical applications of pleuromutilins, coupled with their activity in this intracellular context, highlight their potential as repurposing candidates for anti-*Burkholderia* drug development and thus represent a promising chemotype not previously linked to anti-*Burkholderia* activity. Follow-up studies on these and other novel hits are planned.

Another important outcome of SCRIBE was the identification of host‑directed compounds that impair *Burkholderia* intracellular replication. These findings highlight the potential of host‑directed therapies (HDTs) as adjunctive or complementary approaches to pathogen‑targeted antibiotics. While conventional antimicrobials exert their activity on bacterial processes such as cell wall synthesis or protein translation, HDTs interfere with host cellular factors exploited by *Burkholderia* for cell entry, cytosolic survival, intracellular replication, and cell-to-cell spread. By disrupting host processes (*e.g.,* receptor‑driven signaling, cytoskeletal reorganization, immunomodulatory networks), HDTs compromise intracellular niches that support bacterial persistence and virulence. This host‑focused mode of action also offers the possibility of broad‑spectrum activity, as many intracellular pathogens exploit conserved host processes during infection.

A major advantage of HDTs is the reduced potential for development of antimicrobial resistance. Bacterial resistance to antibiotics typically results from mutations in drug targets under strong selective pressure, whereas host pathways are far less susceptible to such rapid adaptive changes. Consequently, by perturbing host mechanisms rather than bacterial processes, HDTs exert minimal direct selective pressure on the pathogen itself and may substantially delay or mitigate the emergence of resistance. HDTs may also exhibit synergistic effects when used in combination with conventional antibiotics. For example, inhibition of host proteins or metabolic pathways required for intracellular survival could sensitize bacteria to existing antibiotics, potentially overcoming resistance mechanisms and improving treatment efficacy. Such combination strategies are particularly relevant for infections that are difficult to manage with antibiotics alone, such as melioidosis and glanders.

SCRIBE identified 4 host-directed compounds active against *Bp*, 12 against *Bt*, and 21 against *Bm*. This distribution is consistent with the differences in host dependence and pathogenicity between the organisms. *Bp*, which persists in diverse environmental reservoirs and can infect most mammals, yielded the fewest active compounds, consistent with its relative independence from host factors for survival. In contrast, *Bm*, a host-adapted clone of *Bp* with equids as primarily reservoir, showed the largest number of inhibitors which correlates with its reliance on host-interactions for transmission and continued survival as an organism. *Bt* displayed an intermediate profile, consistent with partial overlap in host-interaction mechanisms with *Bp* and *Bm* while lacking in key virulence determinants (and thus increased vulnerability).

Among these host-directed compounds, the protein kinase C (PKC) activator phorbol 12-myristate 13-acetate, impaired intracellular replication across all three *Burkholderia* species (Fig 7). PMA modulates multiple signaling pathways, including PKC, NF-kB and MEK/ERK, and can indirectly activate phospholipase C. These effects impact cellular processes such as growth, differentiation, survival, cytoskeletal dynamics, and adhesion. PKC activation can enhance immune cell functions, including phagocytosis in macrophages and neutrophils, increase reactive oxygen species production to kill intracellular bacteria, modulate autophagy to promote bacterial sequestration in autophagosomes, and affect apoptosis. These activities could alter *Burkholderia*’s ability to enter, survive, replicate, and form MNGCs. Ingenol mebutate, another PKC activator, also exhibited activity against all three species, though the dose-response threshold was only met with *Bt* and *Bm* (Fig 8).

Beyond compound discovery, SCRIBE’s translational relevance was demonstrated in mouse infection studies, where both epetraborole and the identified benchmark fluoroquinolone antibiotic levofloxacin provided complete protection against lethal aerosol challenge with *Bp* and *Bm*. The *in vivo* efficacy of epetraborole was especially compelling, as treatment for 14 days resulted in complete clearance of bacteria from the lungs and spleen across multiple independent experiments and for both virulent *Burkholderia* species (Figs 9-11). These findings align with recent work by Cummings and colleagues, who reported that subcutaneous administration of epetraborole for a period of 3 days post-infection markedly reduced bacterial burdens in the lungs of mice challenged with diverse *Bp* strains [35]. Of note, although trimethoprim and levofloxacin have been previously evaluated against *Burkholderia spp.*, their consistent identification as a top hit across all blinded LOPAC and ReFRAME screens—without prior knowledge of their identity during the screening process—provides independent confirmation of the robustness and predictive power of the SCRIBE platform.

In contrast, the host-directed compound diphenyleneidonium (DPI), which interferes with host oxidative and metabolic pathways [40–44] and inhibited intracellular replication of *Bt*, *Bp* and *Bm in vitro* (Fig 7), failed to confer protection *in vivo*. DPI-treated mice succumbed to lethal *Bp* challenge at rates comparable to placebo-treated controls, and surviving animals showed no reduction in lung or spleen bacterial burdens 3 days post-infection (Fig 9). This divergence between robust *in vitro* activity and lack of *in vivo* efficacy highlights the challenge of advancing host-directed therapeutics: perturbing host pathways hijacked by pathogens for intracellular replication may compromise core immune defense mechanisms or exhibit unacceptable toxicity in whole organisms. Nevertheless, the repeated identification of host-directed compounds across SCRIBE screens suggests that host pathways remain viable targets for future therapeutic development, particularly if more selective or less toxic host-directed compounds, improved pharmacokinetic profiling, and evaluation of combination approaches with direct-acting antibiotics. These steps are intended to better predict *in vivo* success and improve translational success.

The identification of levofloxacin, a known anti-*Burkholderia* agent, throughout our blinded screening of the LOPAC and ReFRAME libraries was not unexpected but provides important validation of the SCRIBE platform. Its consistent detection and the close concordance between our *in vitro* potency data (EC_50_ values and plaque inhibition via the PAS endpoint) and the strong *in vivo* protective efficacy (100% survival and substantial organ clearance) confirm the robustness, accuracy, and predictive power of our high-content imaging assay. This independent confirmation of literature-reported compounds strengthens confidence that other hits identified by SCRIBE, including epetraborole and select host-directed agents, are likewise biologically relevant and translationally promising.

It is important to note that the focus on intracellular replication in SCRIBE is a direct consequence of the assay design and phenotypic readout. The protocol incorporates a kanamycin overlay to eliminate extracellular bacteria, with subsequent quantification of cytopathic effects and monolayer destruction (plaques and syncytia), which result from intracellular replication and cell-to-cell spread. Thus, protection of the monolayer primarily reflects inhibition of intracellular bacterial replication. This does not imply that active compounds lack activity against extracellular bacteria; rather, the endpoint is anchored to the intracellular phase that drives observable CPE in this system. We acknowledge that additional mechanistic studies are needed to more precisely define the stage(s) of the infection cycle impacted by individual compounds (*e.g.,* host cell entry, vacuolar escape, cytosolic replication, or intercellular spread) and to fully elucidate their mode(s) of action. Such studies are ongoing in our laboratory.

While both epetraborole and levofloxacin conferred 100% survival, we noted a modest rebound in spleen bacterial burden in a subset of epetraborole-treated animals between days 14 and 28. This likely reflects the conservative 20 mg/kg dose used, which was constrained by limited compound availability and is well below higher tolerated exposures reported in recent *Bp* studies. Dose escalation, combined with future optimization of treatment initiation time, duration, route (including oral formulations), and dosing regimens, is expected to improve sterilizing activity. Levofloxacin, used here as a benchmark, has demonstrated efficacy in acute murine models but shares known clinical limitations of fluoroquinolones against *Burkholderia spp.*, including potential for relapse due to intracellular persistence and the requirement for prolonged combination therapy in human melioidosis.

Together, our findings establish SCRIBE as a robust, scalable, and phenotypically anchored high-throughput screening platform for identifying small molecules inhibitors of intracellular replication for the Tier 1 Select Agents *Bp* and *Bm,* as well as the BSL-2 surrogate *Bt*. The platform uncovered diverse bioactive chemotypes, including both well-established antibiotic classes and previously unrecognized structural families. The identification of boron‑heterocyclic inhibitors such as epetraborole, which exhibited potent cross‑species activity *in vitro* and conferred complete protection *in vivo* against *Bp* and *Bm*, highlights the platform’s capacity to identify compounds with translational promise. Likewise, the discovery of pleuromutilins and other scaffolds not historically associated with *Burkholderia* broadens the therapeutic chemical space available for countermeasure development. The repeated identification of host‑directed compounds further illustrates the platform’s capacity to reveal modulators of host pathways that shape intracellular persistence, adding an important novel mechanistic dimension to antimicrobial treatment for *Bp* and *Bm*.

An important strength of SCRIBE is its use of Vero E6 cells, a cornerstone of virology, cytopathic-effect assays, and drug discovery. Their well‑characterized morphology, high permissiveness, and compatibility with confocal microscopy enable precise quantification of host‑cell damage, plaque‑like lesions, and monolayer disruption in a high‑throughput format. Because SCRIBE relies on generalizable imaging‑based phenotypes - monolayer integrity, syncytia and plaque formation, and void‑area expansion - the platform can be readily adapted to other mammalian cell types, including those with specialized innate immune functions (*e.g.,* macrophage‑like cells), alternative epithelial cell lines, or primary cells. This flexibility allows researchers to tailor SCRIBE to pathogen‑specific interactions or host‑directed mechanistic studies without altering the underlying analytical workflow.

Beyond *Burkholderia*, the core design elements of SCRIBE - high‑content imaging, modular infection parameters, and phenotype-driven quantification - make the platform broadly applicable to other intracellular bacteria and to viruses that induce cytopathic effects in Vero E6 or other adherent cell lines. Because the PAS readout captures host‑cell damage, plaque formation and cell‑to‑cell spread within a unified framework, SCRIBE provides a versatile experimental foundation for comparative cross‑pathogen drug discovery. Its adaptability positions it as a powerful tool not only for antibacterial screening but also for antiviral discovery and evaluation, host‑pathway interrogation, and the identification of broad‑spectrum agents targeting conserved intracellular vulnerabilities. It should be noted that while the present study focused on *in vivo* validation of select lead compounds, we acknowledge that additional hits identified in the LOPAC and ReFRAME screens warrant further characterization. Future studies will include expanded mechanistic investigations, cytotoxicity profiling, testing in additional relevant cell types (*e.g.,* macrophages), and evaluation of combination therapies for prioritized candidates.

In conclusion, SCRIBE provides a generalizable, high‑resolution framework for discovering therapeutics that disrupt intracellular replication and pathogenesis. Its demonstrated success across *Bp*, *Bm*, and *Bt*, combined with the platform’s flexibility to incorporate diverse cell types and chemotypes, underscores its potential to accelerate countermeasure development against a wide array of high‑consequence intracellular pathogens. Future studies expanding the *in vivo* evaluation of broad-spectrum as well as organism-specific inhibitors of *Burkholderia* intracellular replication, optimization of promising chemotypes (*e.g.,* boron-containing heterocyclic compounds, pleuromutilins), mechanistic studies of novel chemotypes and host-directed compounds, and testing combinatorial treatment strategies will further enhance the translational value of this platform and support the advancement of urgently needed therapeutics for melioidosis, glanders, and beyond.

## Materials and methods

### Ethics statements

The University of Georgia’s Institutional Biosafety Committee (IBC) approved the experiments in this study. All experiments with live *Bm* and *Bp* were performed inside a Class II Biosafety Cabinet (BSC) inside a biosafety level 3 (BSL3) laboratory in compliance with the rules and regulations of the United States Federal Select Agent Program. Infected animals were housed in an Innorack IVC dual-HEPA-filtered ventilated system (Innovive) located in an animal BSL3 (ABSL3) laboratory.

The University of Georgia’s Institutional Animal Care and Use Committee (IACUC) approved the animal experiments in this study. All animal experiments were performed in strict accordance with the recommendations in the Guide for the Care and Use of Laboratory Animals of the National Institutes of Health.

### Bacterial strains, tissue culture cell lines, and growth conditions

The *Burkholderia* strains *B. thailandensis* (*Bt*) DW503 [45], *B. pseudomallei* (*Bp*) K96243 [46] and *B. mallei* (*Bm*) ATCC 23344 [47] were used in the study. *Bt* DW503 and *Bp* K96243 were cultured on tryptic soy agar (BD) for 20 hours at 37^o^C. Plate-grown bacteria were suspended in phosphate-buffered saline (PBS, Corning) to an optical density at a wavelength of 600nm (OD_600_) of 1.0 using a Genesys 30 Visible Spectrophotometer (Thermo Fisher Scientific), which corresponds to a bacterial concentration of 10^8^ colony forming units (CFU) per mL. The suspensions were then serially diluted and used to inoculate cell culture assay plates and/or mice, and 100-µL portions were spread onto agar plates to determine the number of CFU in the inoculum. *Bm* ATCC 23344 was routinely grown on Brucella medium agar (BD) supplemented with glycerol at a final concentration of 5% (vol/vol) for 40 hours at 37^o^C. Plate-grown *Bm* bacteria were treated as described above to prepare inocula and infect cell culture assay plates and mice.

The Vero E6 cell line was purchased from the American Type Culture Collection (catalog number C1008, RRID: CVCL_0575) and routinely cultured at 37^o^C with 5% CO_2_ using Dulbecco’s Modified Eagle Medium (DMEM, Corning) supplemented with 5% fetal bovine serum (FBS, MilliporeSigma). For infection experiments, the cells were seeded into the wells of 384-well plates (Greiner BIO-ONE) using a Biomek NXP automated workstation (Beckman Coulter Life Sciences) in a volume of 40 μL and incubated for 16–20 hours prior to infection with bacteria.

### Reagents for testing compounds in cell culture and mouse infection experiments

Trimethoprim was purchased from Tokyo Chemical Industry Co. Ltd (TCI). Epetraborole and diphenyleneiodonium were purchased from MCE MedChemExpress. Levofloxacin was purchased from WEST-WARD Pharmaceutical/Hikma as a sterile 5 mg/mL solution in 5% dextrose. For cell culture infection experiments, trimethoprim was reconstituted in dimethyl sulfoxide (DMSO, Fisher Scientific) to a concentration of 50 mM, aliquoted, stored at room temperature, and diluted to desired concentrations using cell culture medium. Working stocks were made fresh from concentrated stocks for each experiment. For mouse infection experiments, epetraborole and diphenyleneiodonium were first reconstituted in DMSO to concentrations of 64 mg/mL and 2.67 mg/mL, respectively, stored at -80^o^C, and diluted to desired treatment doses. Thawed aliquots were discarded after each use. The concentrated epetraborole stock was diluted in buffer containing 5% DMSO, 40% Polyethylene Glycol PEG 300 (spectrum Chemical MFG Corp), 5% Tween80 (MP Biomedicals), and 50% sterile saline (Aspen Veterinary Resources LTD). The reconstituted stock of diphenyleneiodonium was diluted in buffer containing 3% DMSO, 40% Polyethylene Glycol PEG 300, 2% Tween80, and 55% sterile saline.

### Small molecule libraries, screening conditions and workflow

The **L**ibrary of **P**harmacologically **A**ctive **C**ompounds (LOPAC, Sigma Aldrich Research Biochemicals, Inc) and the **Re**purposing, **F**ocused **R**escue, and **A**ccelerated **ME**dchem (ReFRAME) library of small molecules were obtained through a collaborative agreement with Calibr-Skaggs, a non-profit drug discovery division of Scripps Research. The LOPAC contains 1,280 compounds for validating drug discovery assays, characterizing orphan receptors, and serves as a reference for drug discovery research. The ReFRAME library is an open-access screening collection of ~13,000 small molecule drugs, > 70% of which have reached clinical development or undergone significant preclinical profiling. The library was built by Calibr-Skaggs to accelerate the identification of potential therapies for human diseases where there is an unmet critical need for treatment options but less commercial motivation for expensive research and development, such as neglected or rare diseases [24]. A publicly accessible data portal (https://reframedb.org) has been created to share the ReFRAME screen hits, promoting further research and maximizing the impact of the ReFRAME screening collection.

The LOPAC and ReFRAME libraries were provided in pre-spotted, single-point, blinded, 384-well plate format. The day prior to screening, plates containing 60 nL of 10 mM compound per well were removed from -80^o^C storage, thawed at room temperature and centrifuged for 10 minutes at a speed of 3,220 x *g*. The drugs were reconstituted by adding 30 μL per well of DMEM supplemented with 2% fetal bovine serum and either 200 μg/mL (experiments with *Bt* DW503) or 2,000 μg/mL (experiments with *Bp* K96243 and *Bm* ATCC 23344) kanamycin using a Biomek NXP automated workstation. The plates were then sealed with adhesive plate foil, centrifuged at a speed of 3,220 x *g* for 2 minutes, bar-coded, and incubated at 37^o^C for 16–20 hours. In tandem, 384-well plates with bar codes matching those of reconstituted compound plates were seeded with Vero E6 cells and processed as outlined above. After overnight incubation, 30 μL of DMEM was removed from the wells of plates seeded with Vero E6 cells using a Biomek NXP automated workstation, leaving 10 μL of medium to overlay monolayers. Afterwards, the cells were inoculated with 5 or 10 μL of bacterial suspension using a 16-channel Finnpipette F2 Pipette (Thermo Fisher Scientific) and incubated in low evaporation square bioassay dishes (Nunc) at 37^o^C with 5% CO_2_. At the indicated time points, a 20 μL volume of reconstituted compounds was added to the infected monolayers of Vero E6 cells using an epMotion 96 semi-automated electronic pipette, and the plates were incubated in low evaporation square bioassay dishes at 37^o^C with 5% CO_2_. At experimental endpoints, paraformaldehyde (Electron Microscopy Sciences) and Hoechst 33342 (Thermo Fisher Scientific) were added to the wells of the cell culture plates to final concentrations of 4% and 10 μg/mL, respectively. After a 15-minute incubation at room temperature, the plates were submerged in fixative solution containing 4% paraformaldehyde and incubated for an additional 20 minutes. Following this, the plates (with wells filled with fixative solution) were sealed with adhesive plate foil (Thermo Fisher Scientific) and incubated for 16 hours at 4^o^C prior to confocal microscopy analysis. LOPAC and ReFRAME compounds showing bioactivity in single-point assays were resupplied by Calibr-Skaggs (limited subset of compounds meeting pre-determined criteria) in pre-spotted, 8-point dose response, 384-well plate format and tested as described above. Compound identifiers were disclosed by Calibr-Skaggs only after potency was confirmed through dose-response testing of putative hits. Half maximal effective concentration (EC_50_) data were deposited in the aforementioned reframedb.org data portal.

Of note, library compounds were screened at a single concentration of 10 µM, selected based on the potency range of the benchmark control trimethoprim (TMP) and standard practices for repurposing libraries. Dedicated cytotoxicity testing of compounds in the absence of infection was not performed. Because the primary readout is protection of the Vero E6 cell monolayer from infection-induced cytopathic effects (CPE) and plaque formation (quantified via PAS), it was assumed within the design of the assay that compounds causing overt host cell toxicity would manifest as monolayer disruption, similar to or worse than the infected DMSO-treated controls. Thus, hits that preserved monolayer integrity were interpreted as effective at limiting intracellular bacterial replication without gross cytotoxicity under the assay conditions.

### Confocal microscopy analysis

Infection assay plates fixed with paraformaldehyde and stained with Hoechst dye were imaged with an ImageXpress Micro Confocal High-Content Imaging System (4X objective, Molecular Devices) and analyzed using a custom-made module created with a MetaXpress software (Molecular Devices). The module was configured to quantify the loss of host cell monolayer integrity as a function of the sum of void area within each well, and this endpoint readout was termed Plaque Area Summed (PAS, units = μm^2^). The PAS accounts for the total summed area of complete monolayer loss (plaque; absence of Hoechst-stained nuclei) as well as incomplete monolayer loss (syncytia/MNGCs; presence of Hoechst-stained nuclei with average fluorescence intensity above that of uninfected control cells). Briefly, all nuclei within the monolayer were identified by the presence of Hoechst stain. A threshold was then applied to eliminate background signal (average intensity of nuclei vs background). A filter mask applying an average intensity threshold for Hoechst fluorescence signal was next applied to eliminate syncytia/MNGCs. Following this, the remaining dead cells and debris were eliminated based on nucleus size. Implementation of these filter masks resulted in the selection of intact monolayers. From this, a monolayer mask (white) was created by applying a pixel grow function to account for cellular area beyond the nucleus. The monolayer mask was next inverted to create a white void area mask function. The white void area mask was then used to calculate the PAS by adding together all void areas within a well. Of note, the PAS calculation includes dead space which is captured due to the position of the lenses and size/shape of the wells. This dead space is consistent throughout all wells and therefore did not affect readout comparisons. This dead space effect accounts for PAS values measured for uninfected control wells. The image analysis module was tested on archived plates from multiple independent infection assays and found to yield reproducible results.

### Mouse infection experiments

Specific pathogen free (SPF) female BALB/c mice (6–8 weeks) were purchased from Inotiv and allowed to acclimate for at least 1 week prior to use. After administration of anesthetic, the mice were inoculated intratracheally using a Microsprayer nebulizing device (PennCentury) as previously published [30]. The infected animals were monitored daily, food and water were provided *ad libitum*, and humane endpoints were strictly observed. Mice exhibiting signs of intermediate to severe discomfort were euthanized in compliance with the AVMA Guidelines for the Euthanasia of Animals. To determine the bacterial burden in the spleen and lungs, individual tissues were collected post-mortem using standard necropsy techniques, placed in gentleMACS M tubes (Miltenyi Biotec) containing 2 mL of PBS, and homogenized with a gentleMACS Dissociator (Miltenyi Biotec). Following this, the homogenates were serially diluted and plated onto agar medium to determine the number of CFU per organ. The median lethal dose (LD_50_) values for *Bm* ATCC 23344 (818 CFU) and *Bp* K96243 (60 CFU) were previously reported by our group [48].

For hit evaluation, doses were selected based on previously published efficacy and tolerability data in murine models, as well as compound availability. For levofloxacin, the 5 mg/kg dose is consistent with regimens previously shown to be effective. For epetraborole, the 20 mg/kg dose was selected as a conservative starting point due to limited compound availability at the time; recent PK/PD studies have shown that higher doses are well tolerated in mice. Levofloxacin was dosed at 5 mg/kg and epetraborole at 20 mg/kg, administered daily by intraperitoneal injection.

### Data analysis

All 384-well infection assay data points were entered into CDD Vault (Collaborative Drug Discovery, Inc), assigned unique drug identifiers, and managed through the software. For single-point assays, compounds were organized based on their ability to reduce PAS compared to the benchmark protection control drug trimethoprim (TMP) and expressed as the extent to which compounds inhibit the formation of plaques (*i.e.,* % protection). Each assay plate included positive (TMP-treated) and negative (untreated) control wells. The PAS calculated from TMP-treated wells was used as the reference point for 100% protection, and the PAS of wells treated with experimental compounds was compared to the mean PAS of TMP-treated wells. Data were normalized using the equation:


$$\begin{array}{c} \%\;Inhibition=\text{100}\times \left(\frac{Raw\;Data-Average\;Negative\;Control}{Average\;Positive\;Control-Average\;Negative\;Control}\right) \end{array}$$

Heat maps representing % protection were then generated based on this comparison. A Z’ factor was also calculated for each assay plate based on the PAS values of positive and negative control wells, and a Z’ value ≥ 0.5 was considered as a highly reproducible and successful experiment. For dose response experiments, the PAS was used to produce dose-response plots with the Levenberg-Marquardt algorithm and calculate the EC_50_ of bioactive compounds. R^2^ values indicate the goodness-of-fit of the sigmoidal dose-response curves to the four-parameter logistic model.

Mouse survival data were analyzed with the Kaplan-Meier method, and the Log-rank Mantel Cox and Gehan-Breslow-Wilcoxon tests were used to perform statistics. Mouse tissue burden data were analyzed using an unpaired, non-parametric Mann-Whitney *t* test. Analysis and graphing of these *in vivo* data were performed using the GraphPad Prism software.

## Supporting information

S1 Table Plate metrics for ReFRAME single-point screen using *Burkholderia thailandensis* strain DW503. PAS = plaque area sum (µm^2^); mean is presented as mean PAS ± standard deviation; collective (n = 38 plates) mean Z’-factor 0.72; collective (n = 38 plates) mean PAS TMP-treated controls 4.56E + 04 ± 2.12E + 04; collective (n = 38 plates) mean PAS untreated controls 6.74E + 06 ± 5.95E + 05.
(DOCX)

S2 Table EC_50_ values of ReFRAME hits protecting against intracellular replication of *Burkholderia thailandensis* DW503. ^a^Compounds were resupplied in 8-point dose response format with concentrations ranging from 0.004572 to 9.986 µM and tested on 2 separate occasions; average EC_50_ values are shown in µM. Compounds with EC_50_ values > 9.99 µM were considered unconfirmed and are not listed. ^b^Average R^2^ values are shown.
(DOCX)

S3 Table Plate metrics for ReFRAME single-point screen using *Burkholderia pseudomallei* strain K96243. Re-Imaged plates: debris from dunking protocol limited analysis of plate images, specifically, DMSO-treated controls were most affected due to artificially increase DAPI signal. Plates were washed, re-imaged and affected controls removed from calculations. ^#^Failed imaging: debris from dunking protocol inhibited analysis of plate images, specifically, DMSO-treated controls which were most affected due to artificially increase DAPI signal. Controls were unresolvable by analysis. Hits were selected manually. PAS = plaque area sum (um^2^); mean is presented as mean PAS ± standard deviation; collective** (n = 36 plates) mean Z’-factor 0.43; collective** (n = 36 plates) mean PAS TMP-treated controls 1.66E + 06 ± 1.30E + 05; collective^**^ (n = 36 plates) mean PAS DMSO-treated controls 8.30E + 06 ± 1.02E + 06; **calculation excludes failed imaging plates.
(DOCX)

S4 Table EC_50_ values of ReFRAME hits protecting against intracellular replication of *Burkholderia pseudomallei* K96243. ^a^Compounds were resupplied in 8-point dose response format with concentrations ranging from 0.00467 to 9.99 µM and tested on 2 separate occasions; average EC_50_ values are shown in µM. Compounds with average EC_50_ values > 9.99 µM were considered unconfirmed and are not listed. ^b^Average R^2^ values are shown.
(DOCX)

S5 Table Plate metrics for ReFRAME single-point screen using *Burkholderia mallei* strain ATCC 23344. PAS = plaque area sum (µm^2^); mean is presented as mean PAS ± standard deviation; collective (n = 39 plates) mean Z’-factor 0.62; collective (n = 39 plates) mean PAS TMP-treated controls 1.90E + 6 ± 2.27E + 05; collective (n = 39 plates) mean PAS untreated controls 8.96E + 06 ± 6.26E + 05.
(DOCX)

S6 Table EC_50_ values of ReFRAME hits protecting against intracellular replication of *Burkholderia mallei* ATCC 23344. ^a^Compounds were resupplied in 8-point dose response format with concentrations ranging from 0.004521 to 10 µM and tested on 2 separate occasions; average EC_50_ values are shown in µM. Compounds with EC_50_ values ≥ 9.99 µM were considered unconfirmed and are not listed. ^b^Average R^2^ values are shown.
(DOCX)
