# Supplementary material for: Discovery of repurposed drugs that disrupt intracellular replication of Burkholderia pseudomallei and Burkholderia mallei and protect against lethal aerosol infection
Source: PLoS Pathog. 2026 Sep 16;22(9):e1014596. doi: 10.1371/journal.ppat.1014596 (PMC13600621; doi:10.1371/journal.ppat.1014596)
Supplement: Supplementary file 1 [file ppat.1014596.s001.docx]

**S1 Table . Plate metrics for ReFRAME single-point screen using *Burkholderia thailandensis* strain DW503.**

| Plate | PAS Z′-factor | Mean PAS TMP-treated controls (n =16) | Mean PAS untreated controls (n =16) |
| --- | --- | --- | --- |
| W1017835 | 0.29 | 6.88E+03 ± 11.9E+03 | 6.30E+06 ± 1.48E+06 |
| W1017837 | 0.8 | 2.85E+03 ± 2.47E+03 | 7.39E+06 ± 4.89E+05 |
| W1017839 | 0.83 | 9.15E+03 ± 14.0E+03 | 6.70E+06 ± 3.64E+05 |
| W1017841 | 0.77 | 23.0E+03 ± 31.1E+03 | 6.55E+06 ± 4.63E+05 |
| W1017843 | 0.76 | 63.0E+03 ± 180E+03 | 6.92E+06 ± 3.77E+05 |
| W1017845 | 0.84 | 6.86E+03 ± 9.24E+03 | 6.68E+06 ± 3.41E+05 |
| W1017847 | 0.38 | 8.04E+03 ± 8.80E+03 | 6.16E+06 ± 1.26E+06 |
| W1017849 | 0.75 | 4.01E+03 ± 4.59E+03 | 6.56E+06 ± 5.46E+05 |
| W1017851 | 0.79 | 8.35E+03 ± 15.0E+03 | 6.22E+06 ± 4.29E+05 |
| W1017853 | 0.76 | 7.12E+03 ± 14.7E+03 | 6.18E+06 ± 4.78E+05 |
| W1017855 | 0.75 | 11.4E+03 ± 13.6E+03 | 5.66E+06 ± 4.49E+05 |
| W1017857 | 0.78 | 2.85E+03 ± 4.16E+03 | 6.37E+06 ± 4.60E+05 |
| W1017859 | 0.76 | 5.73E+03 ± 12.1E+03 | 5.91E+06 ± 4.62E+05 |
| W1017861 | 0.83 | 7.00E+03 ± 6.98E+03 | 5.21E+06 ± 2.96E+05 |
| W1017863 | 0.69 | 15.5E+03 ± 29.6E+03 | 5.93E+06 ± 5.82E+05 |
| W1017865 | 0.66 | 10.2E+03 ± 31.6E+03 | 5.71E+06 ± 6.21E+05 |
| W1017867 | 0.78 | 10.0E+03 ± 32.4E+03 | 5.72E+06 ± 3.77E+05 |
| W1017869 | 0.54 | 14.8E+03 ± 45.7E+03 | 5.74E+06 ± 8.35E+05 |
| W1017871 | 0.78 | 2.84E+03 ± 5.82E+03 | 6.31E+06 ± 4.52E+05 |
| W1017873 | 0.69 | 1.88E+03 ± 2.64E+03 | 5.64E+06 ± 5.82E+05 |
| W1017875 | 0.67 | 1.32E+03 ± 2.28E+03 | 7.05E+06 ± 7.76E+05 |
| W1017877 | 0.85 | 2.82E+03 ± 4.07E+03 | 7.39E+06 ± 3.77E+05 |
| W1017879 | 0.7 | 3.36E+03 ± 4.18E+03 | 7.11E+06 ± 6.99E+05 |
| W1017881 | 0.86 | 2.81E+03 ± 4.26E+03 | 7.73E+06 ± 3.55E+05 |
| W1017883 | 0.87 | 3.15E+03 ± 4.23E+03 | 7.55E+06 ± 3.24E+05 |
| W1017885 | 0.82 | 9.93E+02 ± 1.04E+03 | 7.79E+06 ± 4.57E+05 |
| W1017887 | 0.77 | 9.94E+03 ± 16.2E+03 | 7.51E+06 ± 5.66E+05 |
| W1017889 | 0.83 | 2.72E+03 ± 3.25E+03 | 7.76E+06 ± 4.25E+05 |
| W1017891 | 0.48 | 7.01E+03 ± 7.98E+03 | 6.85E+06 ± 1.17E+06 |
| W1017893 | 0.82 | 1.42E+03 ± 3.40E+03 | 7.18E+06 ± 4.20E+05 |
| W1017895 | 0.23 | 3.99E+03 ± 6.39E+03 | 6.85E+06 ± 1.74E+06 |
| W1017897 | 0.86 | 2.26E+03 ± 2.49E+03 | 7.77E+06 ± 3.56E+05 |
| W1017899 | 0.78 | 23.0E+03 ± 46.1E+03 | 8.05E+06 ± 5.31E+05 |
| W1017901 | 0.68 | 30.7E+03 ± 66.0E+03 | 7.49E+06 ± 7.19E+05 |
| W1017903 | 0.66 | 40.5E+03 ± 98.5E+03 | 6.75E+06 ± 6.58E+05 |
| W1017905 | 0.82 | 3.70E+03 ± 9.34E+03 | 7.49E+06 ± 4.49E+05 |
| W1017907 | 0.71 | 20.2E+03 ± 35.3E+03 | 7.21E+06 ± 6.63E+05 |
| W1017909 | 0.82 | 7.93E+03 ± 15.3E+03 | 7.64E+06 ± 4.38E+05 |

PAS = plaque area sum (µm^2^); mean is presented as mean PAS ± standard deviation; collective (n = 38 plates) mean Z’-factor 0.72; collective (n = 38 plates) mean PAS TMP-treated controls 4.56E+04 ± 2.12E+04; collective (n = 38 plates) mean PAS untreated controls 6.74E+06 ± 5.95E+05
