# Supplementary material for: Discovery of repurposed drugs that disrupt intracellular replication of Burkholderia pseudomallei and Burkholderia mallei and protect against lethal aerosol infection
Source: PLoS Pathog. 2026 Sep 16;22(9):e1014596. doi: 10.1371/journal.ppat.1014596 (PMC13600621; doi:10.1371/journal.ppat.1014596)
Supplement: Supplementary file 2 [file ppat.1014596.s002.docx]

**S2 Table. EC_50_ values of ReFRAME hits protecting against intracellular replication of *Burkholderia thailandensis* DW503.**

| **Compound type** | **Compound name** | **Mechanism of Action** | **^a^EC_50_** | **^b^R^2^** |
| --- | --- | --- | --- | --- |
| **Antibiotics interfering with DNA synthesis** | Brodimoprim | Inhibits dihydropholate reductase (DHFR) | 0.06 | 0.99 |
|  | Metioprim | Inhibits dihydropholate reductase (DHFR) | 0.07 | 0.97 |
|  | Trimethoprim (TMP) | Inhibits dihydropholate reductase (DHFR) | 0.07 | 0.97 |
|  | 1954U89 | Inhibits dihydropholate reductase (DHFR) | 0.08 | 0.77 |
|  | Iclaprim | Inhibits dihydropholate reductase (DHFR) | 0.12 | 0.99 |
|  | Ormetoprim | Inhibits dihydropholate reductase (DHFR) | 0.20 | 0.99 |
|  | 2,4-Diamino-5-(3,4-Dimethoxybenzyl) Pyrimidine | Inhibits dihydropholate reductase (DHFR) | 0.26 | 0.99 |
|  | DAMP-ES | Inhibits dihydropholate reductase (DHFR) | 0.69 | 0.91 |
|  | Trimetrexate | Inhibits dihydropholate reductase (DHFR) | 0.76 | 0.99 |
|  | Tetroxoprim | Inhibits dihydropholate reductase (DHFR) | 0.92 | 0.96 |
|  | Piritrexim | Inhibits dihydropholate reductase (DHFR) | 0.94 | 0.99 |
|  | Baquiloprim | Inhibits dihydropholate reductase (DHFR) | 1.63 | 0.96 |
|  | Sulfaphenazole | Sulfonamide-class; inhibits dihydropteroate synthase (DHPS) | 0.93 | 0.99 |
|  | Phthalylsulfathiazole | Sulfonamide-class; inhibits dihydropteroate synthase (DHPS) | 0.95 | 0.99 |
|  | Sulfadoxine | Sulfonamide-class; inhibits dihydropteroate synthase (DHPS) | 1.00 | 0.99 |
|  | Sulfamethylthiazole | Sulfonamide-class; inhibits dihydropteroate synthase (DHPS) | 1.00 | 0.99 |
|  | RFM-003-805-1 | Sulfonamide-class; inhibits dihydropteroate synthase (DHPS) | 1.01 | 0.98 |
|  | Sulfalene | Sulfonamide-class; inhibits dihydropteroate synthase (DHPS) | 1.04 | 0.92 |
|  | Sulfametrole | Sulfonamide-class; inhibits dihydropteroate synthase (DHPS) | 1.24 | 0.99 |
|  | Sulfamethoxazole | Sulfonamide-class; inhibits dihydropteroate synthase (DHPS) | 1.26 | 0.99 |
|  | Sulfazamet | Sulfonamide-class; inhibits dihydropteroate synthase (DHPS) | 1.37 | 0.94 |
|  | Sulfamerazine | Sulfonamide-class; inhibits dihydropteroate synthase (DHPS) | 1.57 | 0.99 |
|  | Sulfathiazole | Sulfonamide-class; inhibits dihydropteroate synthase (DHPS) | 1.71 | 0.99 |
|  | Sulfachloropyrazine | Sulfonamide-class; inhibits dihydropteroate synthase (DHPS) | 1.98 | 0.97 |
|  | Sulfaperine | Sulfonamide-class; inhibits dihydropteroate synthase (DHPS) | 2.01 | 0.98 |
|  | Sulfamethomidine | Sulfonamide-class; inhibits dihydropteroate synthase (DHPS) | 2.03 | 0.95 |
|  | Sulfadiazine | Sulfonamide-class; inhibits dihydropteroate synthase (DHPS) | 2.04 | 0.99 |
|  | Sulfapyridine | Sulfonamide-class; inhibits dihydropteroate synthase (DHPS) | 2.27 | 0.98 |
|  | Sulfameter | Sulfonamide-class; inhibits dihydropteroate synthase (DHPS) | 2.36 | 0.94 |
|  | Sulfisoxazole | Sulfonamide-class; inhibits dihydropteroate synthase (DHPS) | 2.38 | 0.99 |
|  | Sulfasomizole | Sulfonamide-class; inhibits dihydropteroate synthase (DHPS) | 3.01 | 0.95 |
|  | Sulfaethiodole | Sulfonamide-class; inhibits dihydropteroate synthase (DHPS) | 3.11 | 0.97 |
|  | Sulfatroxazole | Sulfonamide-class; inhibits dihydropteroate synthase (DHPS) | 3.36 | 0.98 |
|  | Sulfamethoxypyrazine | Sulfonamide-class; inhibits dihydropteroate synthase (DHPS) | 3.98 | 0.99 |
|  | Sulfamonomethoxine | Sulfonamide-class; inhibits dihydropteroate synthase (DHPS) | 5.48 | 0.89 |
|  | Sulfachloropyridazine | Sulfonamide-class; inhibits dihydropteroate synthase (DHPS) | 6.04 | 0.98 |
|  | Sulfisomidine | Sulfonamide-class; inhibits dihydropteroate synthase (DHPS) | 6.52 | 0.99 |
| **Antibiotics interfering with protein synthesis** | Demeclocycline | Tetracycline-class; binds 30S subunit of bacterial ribosome and hinders binding of tRNA | 0.24 | 0.99 |
|  | Nitrocycline | Tetracycline-class; binds 30S subunit of bacterial ribosome and hinders binding of tRNA | 0.24 | 0.99 |
|  | Doxycycline | Tetracycline-class; binds 30S subunit of bacterial ribosome and hinders binding of tRNA | 0.38 | 0.99 |
|  | Minocycline | Tetracycline-class; binds 30S subunit of bacterial ribosome and hinders binding of tRNA | 0.47 | 0.99 |
|  | Methacycline | Tetracycline-class; binds 30S subunit of bacterial ribosome and hinders binding of tRNA | 0.59 | 0.98 |
|  | Meclocycline | Tetracycline-class; binds 30S subunit of bacterial ribosome and hinders binding of tRNA | 0.63 | 0.99 |
|  | Incyclinide | Tetracycline-class; binds 30S subunit of bacterial ribosome and hinders binding of tRNA | 0.73 | 0.97 |
|  | RFM-001-392-3 | Tetracycline-class; binds 30S subunit of bacterial ribosome and hinders binding of tRNA | 0.75 | 0.99 |
|  | Omadacycline | Tetracycline-class; binds 30S subunit of bacterial ribosome and hinders binding of tRNA | 1.62 | 0.99 |
|  | Eravacycline | Tetracycline-class; binds 30S subunit of bacterial ribosome and hinders binding of tRNA | 5.46 | 0.99 |
|  | Chlortetracycline | Tetracycline-class; binds 30S subunit of bacterial ribosome and hinders binding of tRNA | 8.55 | 0.98 |
|  | Chloramphenicol | Chloramphenicol-class; binds 50S subunit of bacterial ribosome and inhibits the action of peptidyl transferase | 0.85 | 0.99 |
|  | Florfenicol | Chloramphenicol-class; binds 50S subunit of bacterial ribosome and inhibits the action of peptidyl transferase | 1.61 | 0.99 |
|  | Valnemulin | Pleuromutilin-class; binds 50S subunit of bacterial ribosome and inhibits the action of peptidyl transferase | 2.27 | 0.99 |
|  | BC-3205 | Pleuromutilin-class; binds 50S subunit of bacterial ribosome and inhibits the action of peptidyl transferase | 3.24 | 0.98 |
|  | SB-268091 | Pleuromutilin-class; binds 50S subunit of bacterial ribosome and inhibits the action of peptidyl transferase | 7.99 | 0.89 |
|  | Tiamulin | Pleuromutilin-class; binds 50S subunit of bacterial ribosome and inhibits the action of peptidyl transferase | 9.70 | 0.95 |
|  | HMR-3004 | Macrolide-class; binds 50S subunit of bacterial ribosome and inhibits the action of peptidyl transferase | 3.38 | 0.99 |
|  | GBB-83698 | Inhibits activity of bacterial peptide deformylase (PDF) | 3.31 | 0.98 |
|  | GSK-1322322 | Inhibits activity of bacterial peptide deformylase (PDF) | 7.03 | 0.99 |
|  | Epetraborole | Benzoxaborole-class; boron-heterocyclic compound that targets bacterial leucyl-tRNA synthase (LeuRS) | 1.11 | 0.97 |
|  | RFM-007-075-7 | Benzoxaborole-class; boron-heterocyclic compound that targets bacterial leucyl-tRNA synthase (LeuRS) | 1.61 | 0.98 |
| **Antibiotics interfering with RNA synthesis** | LM-565 | Rifamycin-class; binds 𝛃 subunit of bacterial RNA polymerase and inhibits synthesis of RNA | 1.06 | 0.96 |
|  | Mycobutin | Rifamycin-class; binds 𝛃 subunit of bacterial RNA polymerase and inhibits synthesis of RNA | 2.94 | 0.98 |
|  | Rifapentine | Rifamycin-class; binds 𝛃 subunit of bacterial RNA polymerase and inhibits synthesis of RNA | 4.03 | 0.98 |
|  | 9-Aminoacridine | Inhibits ribosome biogenesis; targets transcription and processing of ribosomal RNA, used as antiseptic | 9.97 | 0.97 |
| **Antibiotics interfering with fatty acid synthesis** | Debio-1453 | Inhibits activity of bacterial FabI enoyl-acyl carrier protein (ACP) reductase | 1.92 | 0.98 |
| **Antibiotics interfering with synthesis of lipopolysaccharides** | ACHN-975 | Inhibits activity of bacterial Uridine diphosphate-3-O-(hydroxymyristoyl)-N-acetylglucosamine deacetylase LpxC in Gram-negative bacteria | 0.95 | 0.95 |
| **Antibiotics interfering with DNA replication** | DC-159a | Fluoroquinolone-class; inhibits DNA gyrase | 0.03 | 0.99 |
|  | RFM-011-104-6 | Fluoroquinolone-class; inhibits DNA gyrase | 0.03 | 1.00 |
|  | DK-507K | Fluoroquinolone-class; inhibits DNA gyrase | 0.04 | 0.99 |
|  | Danofloxacin | Fluoroquinolone-class; inhibits DNA gyrase | 0.04 | 0.99 |
|  | Sitafloxacin | Fluoroquinolone-class; inhibits DNA gyrase | 0.04 | 0.99 |
|  | Gatifloxacin | Fluoroquinolone-class; inhibits DNA gyrase | 0.05 | 0.99 |
|  | PD-131112 | Fluoroquinolone-class; inhibits DNA gyrase | 0.05 | 0.99 |
|  | BMY-41802 | Fluoroquinolone-class; inhibits DNA gyrase | 0.05 | 0.97 |
|  | Temafoxacin | Fluoroquinolone-class; inhibits DNA gyrase | 0.06 | 0.99 |
|  | Clinafloxacin | Fluoroquinolone-class; inhibits DNA gyrase | 0.07 | 0.99 |
|  | BMY-43748 | Fluoroquinolone-class; inhibits DNA gyrase | 0.08 | 0.99 |
|  | Tosufloxacin | Fluoroquinolone-class; inhibits DNA gyrase | 0.09 | 0.99 |
|  | Nadifloxacin | Fluoroquinolone-class; inhibits DNA gyrase | 0.09 | 0.99 |
|  | Sarafloxacin | Fluoroquinolone-class; inhibits DNA gyrase | 0.13 | 0.99 |
|  | Grepafloxacin | Fluoroquinolone-class; inhibits DNA gyrase | 0.13 | 0.97 |
|  | Cadrofloxacin | Fluoroquinolone-class; inhibits DNA gyrase | 0.14 | 0.99 |
|  | RFM-009-500-1 | Fluoroquinolone-class; inhibits DNA gyrase | 0.14 | 0.98 |
|  | KRQ-10071 | Fluoroquinolone-class; inhibits DNA gyrase | 0.15 | 0.99 |
|  | E-4904 | Fluoroquinolone-class; inhibits DNA gyrase | 0.16 | 0.99 |
|  | BMY-40062 | Fluoroquinolone-class; inhibits DNA gyrase | 0.16 | 0.98 |
|  | E-5065 | Fluoroquinolone-class; inhibits DNA gyrase | 0.16 | 0.99 |
|  | Y-688 | Fluoroquinolone-class; inhibits DNA gyrase | 0.16 | 0.99 |
|  | E-4884 | Fluoroquinolone-class; inhibits DNA gyrase | 0.17 | 0.98 |
|  | Enrofloxacin | Fluoroquinolone-class; inhibits DNA gyrase | 0.17 | 0.99 |
|  | Finafloxacin | Fluoroquinolone-class; inhibits DNA gyrase | 0.19 | 0.99 |
|  | WCK-1152 | Fluoroquinolone-class; inhibits DNA gyrase | 0.19 | 0.98 |
|  | CP-74667 | Fluoroquinolone-class; inhibits DNA gyrase | 0.19 | 0.99 |
|  | Delafloxacin | Fluoroquinolone-class; inhibits DNA gyrase | 0.20 | 0.98 |
|  | Pefloxacin | Fluoroquinolone-class; inhibits DNA gyrase | 0.20 | 0.99 |
|  | Protosufloxacin | Fluoroquinolone-class; inhibits DNA gyrase | 0.27 | 0.99 |
|  | Moxifloxacin | Fluoroquinolone-class; inhibits DNA gyrase | 0.28 | 0.99 |
|  | KPI-10 | Fluoroquinolone-class; inhibits DNA gyrase | 0.29 | 0.99 |
|  | RFM-003-154-9 | Fluoroquinolone-class; inhibits DNA gyrase | 0.29 | 0.99 |
|  | A-57132, RFM-009-978-5 | Fluoroquinolone-class; inhibits DNA gyrase | 0.29 | 0.99 |
|  | Levofloxacin | Fluoroquinolone-class; inhibits DNA gyrase | 0.30 | 0.99 |
|  | DW-286 | Fluoroquinolone-class; inhibits DNA gyrase | 0.34 | 0.98 |
|  | WQ-2756 | Fluoroquinolone-class; inhibits DNA gyrase | 0.37 | 0.98 |
|  | WCK-2349 (Alalevonadifloxacin) | Fluoroquinolone-class; inhibits DNA gyrase | 0.49 | 0.98 |
|  | Sparfloxacin | Fluoroquinolone-class; inhibits DNA gyrase | 0.50 | 0.99 |
|  | Rufloxacin | Fluoroquinolone-class; inhibits DNA gyrase | 0.51 | 0.99 |
|  | Gemifloxacin | Fluoroquinolone-class; inhibits DNA gyrase | 0.53 | 0.99 |
|  | Alatrofloxacin | Fluoroquinolone-class; inhibits DNA gyrase | 0.57 | 0.99 |
|  | Ibafloxacin | Fluoroquinolone-class; inhibits DNA gyrase | 0.59 | 0.77 |
|  | Pazufloxacin | Fluoroquinolone-class; inhibits DNA gyrase | 0.59 | 0.99 |
|  | Marbofloxacin | Fluoroquinolone-class; inhibits DNA gyrase | 0.64 | 0.99 |
|  | Enofloxacin | Fluoroquinolone-class; inhibits DNA gyrase | 0.65 | 0.99 |
|  | CI-990 | Fluoroquinolone-class; inhibits DNA gyrase | 0.65 | 0.99 |
|  | R-71762 | Fluoroquinolone-class; inhibits DNA gyrase | 0.66 | 0.95 |
|  | OPC-7241 (Vebufloxacin) | Fluoroquinolone-class; inhibits DNA gyrase | 0.74 | 0.97 |
|  | Bay-y-3118 | Fluoroquinolone-class; inhibits DNA gyrase | 0.79 | 0.99 |
|  | WIN-57273 | Fluoroquinolone-class; inhibits DNA gyrase | 0.86 | 0.99 |
|  | S-31076 | Fluoroquinolone-class; inhibits DNA gyrase | 0.95 | 0.98 |
|  | Flumequine | Fluoroquinolone-class; inhibits DNA gyrase | 0.96 | 0.99 |
|  | Ulifloxacin | Fluoroquinolone-class; inhibits DNA gyrase | 1.03 | 0.99 |
|  | Ecenofloxacin | Fluoroquinolone-class; inhibits DNA gyrase | 1.07 | 0.94 |
|  | AZD-9742 | Fluoroquinolone-class; inhibits DNA gyrase | 1.34 | 0.83 |
|  | Prulifloxacin | Fluoroquinolone-class; inhibits DNA gyrase | 1.56 | 0.99 |
|  | Ciprofloxacin | Fluoroquinolone-class; inhibits DNA gyrase | 1.67 | 0.99 |
|  | WQ-1197 | Fluoroquinolone-class; inhibits DNA gyrase | 2.15 | 0.98 |
|  | Balofloxacin | Fluoroquinolone-class; inhibits DNA gyrase | 2.17 | 0.99 |
|  | AZD-0914 (Zolifodacin) | Fluoroquinolone-class; inhibits DNA gyrase | 2.75 | 0.98 |
|  | Orbifloxacin | Fluoroquinolone-class; inhibits DNA gyrase | 2.81 | 0.99 |
|  | JNJ-Q2 | Fluoroquinolone-class; inhibits DNA gyrase | 3.36 | 0.97 |
|  | RFM-010-096-9 | Fluoroquinolone-class; inhibits DNA gyrase | 4.85 | 0.91 |
|  | Olamufloxacin | Fluoroquinolone-class; inhibits DNA gyrase | 5.01 | 0.99 |
|  | DV-7751A | Fluoroquinolone-class; inhibits DNA gyrase | 5.14 | 0.93 |
|  | E-4767 | Fluoroquinolone-class; inhibits DNA gyrase | 5.23 | 0.99 |
|  | Besifloxacin | Fluoroquinolone-class; inhibits DNA gyrase | 5.51 | 0.96 |
|  | A-83669 | Fluoroquinolone-class; inhibits DNA gyrase | 7.21 | 0.98 |
|  | Nemonoxacin | Quinolone-class; inhibits DNA gyrase | 0.10 | 0.98 |
|  | Garenoxacin | Quinolone-class; inhibits DNA gyrase | 0.11 | 0.99 |
|  | PGE-9509924 | Quinolone-class; inhibits DNA gyrase | 0.14 | 0.99 |
|  | Rosoxacin | Quinolone-class; inhibits DNA gyrase | 0.25 | 0.97 |
|  | Irloxacin | Quinolone-class; inhibits DNA gyrase | 0.30 | 0.99 |
|  | T-3912 | Quinolone-class; inhibits DNA gyrase | 0.46 | 0.99 |
|  | PGE-4175997 | Quinolone-class; inhibits DNA gyrase | 0.59 | 0.99 |
|  | PD-161314 | Quinolone-class; inhibits DNA gyrase | 0.76 | 0.96 |
|  | DJ-6783 | Quinolone-class; inhibits DNA gyrase | 2.53 | 0.99 |
|  | Miloxacin | Quinolone-class; inhibits DNA gyrase | 6.49 | 0.93 |
|  | Oxolinic acid | Quinolone-class; inhibits DNA gyrase | 8.31 | 0.99 |
| **Host-directed compounds** | Phorbol 12-myristate 13-acetate | Protein kinase C activator; stimulates T-cell activation and cytokine production | 0.08 | 0.99 |
|  | Ingenol mebutate | Protein kinase C activator; causes cell death via necrosis | 0.12 | 0.97 |
|  | Resiniferatoxin | Transient Vanilloid VR1 Receptor TRPV1 agonist | 1.41 | 0.99 |
|  | Naftazone | Increases venous tonicity and capillary resistance, reduces aggregation of platelets and activation/adhesion of leukocytes | 6.39 | 0.77 |
|  | Calcium Bromide | Functions as sedative and anticonvulsant by modulating neuronal activity | 6.61 | 0.91 |
|  | Triamterene | Potassium-sparing diuretic; blocks epithelial sodium channel (ENaC) on the lumen side of the kidney collecting tubule. | 6.97 | 0.81 |
|  | Belotecan | Camptothecin derivative; anti-cancer drug that inhibits activity of DNA topoisomerase I | 7.05 | 0.85 |
|  | Simmitecan | Camptothecin derivative; anti-cancer drug that inhibits activity of DNA topoisomerase I | 8.15 | 0.97 |
|  | Irinotecan | Camptothecin derivative; anti-cancer drug that inhibits activity of DNA topoisomerase I | 9.90 | 0.99 |
|  | OSI-211 (Lurtotecan) | Camptothecin derivative; anti-cancer drug that inhibits activity of DNA topoisomerase I | 9.91 | 0.89 |
| **Not disclosed due to proprietary restrictions** | CBR-001-624-964-2 | Not available | 0.26 | 0.99 |
|  | CBR-001-572-455-7 | Not available | 0.37 | 0.99 |
|  | CBR-001-635-898-8 | Not available | 2.37 | 0.97 |
|  | CBR-001-625-519-9 | Not available | 5.06 | 0.67 |
|  | CBR-001-593-053-7 | Not available | 7.05 | 0.99 |
|  | CBR-001-584-067-2 | Not available | 8.26 | 0.95 |

^a^Compounds were resupplied in 8-point dose response format with concentrations ranging from 0.004572 to 9.986 µM and tested on 2 separate occasions; average EC_50_ values are shown in µM. Compounds with EC_50_ values > 9.99 µM were considered unconfirmed and are not listed.

^b^Average R^2^ values are shown.
