# Supplementary material for: Discovery of repurposed drugs that disrupt intracellular replication of Burkholderia pseudomallei and Burkholderia mallei and protect against lethal aerosol infection
Source: PLoS Pathog. 2026 Sep 16;22(9):e1014596. doi: 10.1371/journal.ppat.1014596 (PMC13600621; doi:10.1371/journal.ppat.1014596)
Supplement: Supplementary file 3 [file ppat.1014596.s003.docx]

**S3 Table. Plate metrics for ReFRAME single-point screen using *Burkholderia pseudomallei* strain K96243.**

| Plate | PAS Z′-factor | Mean PAS TMP-treated controls (n =16 wells per plate) | Mean PAS untreated controls (n =16 wells per plate) |
| --- | --- | --- | --- |
| W1022953 | 0.72 | 1.83E+06 ± 119E+03 | 10.7E+06 ± 695E+03 |
| W1022955 | 0.68 | 1.88E+06 ± 133E+03 | 10.4E+06 ± 779E+03 |
| W1022957 | 0.7 | 1.86E+06 ± 122E+03 | 10.5E+06 ± 753E+03 |
| W1022959 | 0.84 | 1.87E+06 ± 101E+03 | 10.8E+06 ± 381E+03 |
| W1022961 | 0.63 | 1.77E+06 ± 83.5E+03 | 9.57E+06 ± 886E+03 |
| W1022963 | 0.46 | 1.78E+06 ± 96.7E+03 | 9.46E+06 ± 1.29E+06 |
| W1022965 | 0.28 | 1.77E+06 ± 154E+03 | 8.27E+06 ± 1.41E+06 |
| W1022967 | 0.47 | 1.75E+06 ± 251E+03 | 8.69E+06 ± 971E+03 |
| W1022971 | 0.37 | 1.66E+06 ± 180E+03 | 8.49E+06 ± 1.24E+06 |
| W1022973 | 0.38 | 1.79E+06 ± 345E+03 | 9.03E+06 ± 1.14E+06 |
| W1022977 | 0.41 | 1.59E+06 ± 171E+03 | 8.09E+06 ± 1.10E+06 |
| W1022979 | 0.47 | 1.91E+06 ± 223E+03 | 9.35E+06 ± 1.09E+06 |
| W1022981 | 0.45 | 1.57E+06 ± 113E+03 | 8.59E+06 ± 1.17E+06 |
| W1022983 | 0.58 | 1.77E+06 ± 68.5E+03 | 9.53E+06 ± 1.03E+06 |
| W1022995 | 0.32 | 1.71E+06 ± 102E+03 | 9.32E+06 ± 1.62E+06 |
| W1023001 | 0.55 | 1.68E+06 ± 103E+03 | 9.83E+06 ± 1.11E+06 |
| W1023003 | 0.35 | 1.61E+06 ± 77.5E+03 | 8.88E+06 ± 1.50E+06 |
| W1023005 | 0.59 | 1.64E+06 ± 83.5E+03 | 9.92E+06 ± 1.04E+06 |
| W1023007 | 0.34 | 1.60E+06 ± 81.2E+03 | 7.99E+06 ± 1.33E+06 |
| W1023017 | 0.64 | 1.73E+06 ± 64.6E+03 | 10.1E+06 ± 948E+03 |
| W1023019 | 0.33 | 1.71E+06 ± 125E+03 | 9.00E+06 ± 1.51E+06 |
| W1023021 | 0.68 | 1.65E+06 ± 107E+03 | 10.3E+06 ± 814E+03 |
| W1023023 | 0.32 | 1.63E+06 ± 93.0E+03 | 8.94E+06 ± 1.57E+06 |
| W1023025 | 0.64 | 1.68E+06 ± 104E+03 | 10.7E+06 ± 964E+03 |
| W1023029 | 0.62 | 1.49E+06 ± 108E+03 | 9.71E+06 ± 946E+03 |
| W1024565* | 0.69 | 1.68E+06 ± 340E+03 | 8.96E+06 ± 416E+03 |
| W1022989* | 0.04 | 1.45E+06 ± 138E+03 | 4.95E+06 ± 986E+03 |
| W1022987* | 0.1 | 1.46E+06 ± 112E+03 | 5.08E+06 ± 978E+03 |
| W1022969* | 0.25 | 1.48E+06 ± 120E+03 | 4.46E+06 ± 622E+03 |
| W1023011* | 0.34 | 1.44E+06 ± 117E+03 | 5.26E+06 ± 717E+03 |
| W1022975* | 0.33 | 1.49E+06 ± 111E+03 | 4.87E+06 ± 638E+03 |
| W1023013* | 0.15 | 1.44E+06 ± 112E+03 | 5.16E+06 ± 947E+03 |
| W1023009* | 0.21 | 1.44E+06 ± 127E+03 | 5.71E+06 ± 1.00E+06 |
| W1022997* | 0.18 | 1.60E+06 ± 72.0E+03 | 5.09E+06 ± 877E+03 |
| W1023027* | 0.17 | 1.61E+06 ± 60.9E+03 | 9.07E+06 ± 2.00E+06 |
| W1022993* | 0.31 | 1.58E+06 ± 152E+03 | 3.88E+06 ± 377E+03 |
| W1022985^#^ | -0.51 | 1.58E+06 ± 102E+03 | 8.32E+06 ± 3.30E+06 |
| W1022991^#^ | -0.05 | 1.79E+06 ± 110E+03 | 3.65E+06 ± 541E+03 |
| W1022999^#^ | 0.17 | 1.58E+06 ± 152E+03 | 3.79E+06 ± 458E+03 |

Re-Imaged plates: debris from dunking protocol limited analysis of plate images, specifically, DMSO-treated controls were most affected due to artificially increase DAPI signal. Plates were washed, re-imaged and affected controls removed from calculations.

^#^Failed imaging: debris from dunking protocol inhibited analysis of plate images, specifically, DMSO-treated controls which were most affected due to artificially increase DAPI signal. Control were unresolvable by analysis. Hits were selected manually.

PAS = plaque area sum (um^2^); mean is presented as mean PAS ± standard deviation; collective** (n = 36 plates) mean Z’-factor 0.43; collective** (n = 36 plates) mean PAS TMP-treated controls 1.66E+06 ± 1.30E+05; collective^**^ (n = 36 plates) mean PAS DMSO-treated controls 8.30E+06 ± 1.02E+06; **calculation excludes failed imaging plates
