# Supplementary material for: Discovery of repurposed drugs that disrupt intracellular replication of Burkholderia pseudomallei and Burkholderia mallei and protect against lethal aerosol infection
Source: PLoS Pathog. 2026 Sep 16;22(9):e1014596. doi: 10.1371/journal.ppat.1014596 (PMC13600621; doi:10.1371/journal.ppat.1014596)
Supplement: Supplementary file 4 [file ppat.1014596.s004.docx]

**S4 Table. EC_50_ values of ReFRAME hits protecting against intracellular replication of *Burkholderia pseudomallei* K96243.**

| **Compound type** | Compound name | Mechanism of Action | ^a^EC_50_ | ^b^R^2^ |
| --- | --- | --- | --- | --- |
| **Antibiotics interfering with DNA synthesis** | Brodimoprim | Inhibits dihydropholate reductase (DHFR) | 0.33 | 0.96 |
|  | Iclaprim | Inhibits dihydropholate reductase (DHFR) | 0.38 | 0.98 |
|  | Metioprim | Inhibits dihydropholate reductase (DHFR | 0.63 | 0.99 |
|  | 1954U89 | Inhibits dihydropholate reductase (DHFR) | 0.91 | 0.98 |
|  | Ormetoprim | Inhibits dihydropholate reductase (DHFR) | 1.98 | 0.99 |
|  | 2,4-Diamino-5-(3,4-Dimethoxybenzyl) Pyrimidine | Inhibits dihydropholate reductase (DHFR) | 2.22 | 0.93 |
|  | Aditoprim | Inhibits dihydropholate reductase (DHFR) | 3.23 | 0.99 |
|  | Sulfathiazole | Sulfonamide-class; inhibits dihydropteroate synthase (DHPS) | 0.41 | 0.99 |
|  | Sulfamethoxazole | Sulfonamide-class; inhibits dihydropteroate synthase (DHPS) | 0.53 | 0.99 |
|  | Sulfamethizole | Sulfonamide-class; inhibits dihydropteroate synthase (DHPS) | 0.53 | 0.93 |
|  | Sulfachloropyrazine | Sulfonamide-class; inhibits dihydropteroate synthase (DHPS) | 0.96 | 0.89 |
|  | Sulfaethiodole | Sulfonamide-class; inhibits dihydropteroate synthase (DHPS) | 1.41 | 0.99 |
|  | Sulfamethylthiazole | Sulfonamide-class; inhibits dihydropteroate synthase (DHPS) | 1.65 | 0.99 |
|  | Sulfachloropyridazine | Sulfonamide-class; inhibits dihydropteroate synthase (DHPS) | 1.70 | 0.99 |
|  | Sulfameter | Sulfonamide-class; inhibits dihydropteroate synthase (DHPS) | 2.58 | 0.98 |
|  | Sulfasomizole | Sulfonamide-class; inhibits dihydropteroate synthase (DHPS) | 2.90 | 0.88 |
|  | Phthalylsulfathiazole | Sulfonamide-class; inhibits dihydropteroate synthase (DHPS) | 3.05 | 0.86 |
|  | Sulfalene | Sulfonamide-class; inhibits dihydropteroate synthase (DHPS) | 3.14 | 0.99 |
|  | Sulfadiazine | Sulfonamide-class; inhibits dihydropteroate synthase (DHPS) | 3.49 | 0.63 |
|  | Sulfamonomethoxine | Sulfonamide-class; inhibits dihydropteroate synthase (DHPS) | 4.90 | 0.99 |
|  | RFM-003-805-1 | Sulfonamide-class; inhibits dihydropteroate synthase (DHPS) | 5.17 | 0.96 |
|  | Sulfaphenazole | Sulfonamide-class; inhibits dihydropteroate synthase (DHPS) | 5.39 | 0.86 |
|  | Sulfametrole | Sulfonamide-class; inhibits dihydropteroate synthase (DHPS) | 5.41 | 0.95 |
|  | Sulfamerazine | Sulfonamide-class; inhibits dihydropteroate synthase (DHPS) | 5.47 | 0.99 |
|  | Sulfisoxazole | Sulfonamide-class; inhibits dihydropteroate synthase (DHPS) | 5.74 | 0.97 |
|  | Sulfadicramide | Sulfonamide-class; inhibits dihydropteroate synthase (DHPS) | 5.19 | 0.81 |
|  | Sulfadimethoxine | Sulfonamide-class; inhibits dihydropteroate synthase (DHPS) | 6.12 | 0.78 |
|  | Sulfaperine | Sulfonamide-class; inhibits dihydropteroate synthase (DHPS) | 9.99 | 0.93 |
|  | Azanidazole | Nitroimidazole-class; causes DNA damage | 3.60 | 0.89 |
| **Antibiotics interfering with protein synthesis** | Nitrocycline | Tetracycline-class; binds 30S subunit of bacterial ribosome and hinders binding of tRNA | 0.44 | 0.99 |
|  | Doxycycline | Tetracycline-class; binds 30S subunit of bacterial ribosome and hinders binding of tRNA | 0.51 | 0.99 |
|  | Demeclocycline | Tetracycline-class; binds 30S subunit of bacterial ribosome and hinders binding of tRNA | 1.07 | 0.94 |
|  | Epetraborole | Benzoxaborole-class; boron-heterocyclic compound that targets bacterial leucyl-tRNA synthase (LeuRS) | 1.92 | 0.99 |
| **Antibiotics interfering with fatty acid synthesis** | Debio-1453 | Inhibits activity of bacterial FabI enoyl-acyl carrier protein (ACP) reductase | 1.07 | 0.99 |
| **Antibiotics interfering with DNA replication** | E-3846 | Fluoroquinolone-class; inhibits DNA gyrase | 0.02 | 0.99 |
|  | Nadifloxacin | Fluoroquinolone-class; inhibits DNA gyrase | 0.03 | 0.99 |
|  | Olamufloxacin | Fluoroquinolone-class; inhibits DNA gyrase | 0.03 | 0.99 |
|  | Bay-y-3118 | Fluoroquinolone-class; inhibits DNA gyrase | 0.04 | 0.99 |
|  | S-31076 | Fluoroquinolone-class; inhibits DNA gyrase | 0.05 | 0.99 |
|  | Delafloxacin | Fluoroquinolone-class; inhibits DNA gyrase | 0.05 | 0.99 |
|  | WCK-2349 (Alalevonadifloxacin) | Fluoroquinolone-class; inhibits DNA gyrase | 0.06 | 0.99 |
|  | Sitafloxacin | Fluoroquinolone-class; inhibits DNA gyrase | 0.07 | 0.98 |
|  | Ibafloxacin | Fluoroquinolone-class; inhibits DNA gyrase | 0.08 | 0.99 |
|  | Enrofloxacin | Fluoroquinolone-class; inhibits DNA gyrase | 0.09 | 0.99 |
|  | RFM-009-500-1 | Fluoroquinolone-class; inhibits DNA gyrase | 0.09 | 0.99 |
|  | RFM-011-104-6 | Fluoroquinolone-class; inhibits DNA gyrase | 0.09 | 0.99 |
|  | Alatrofloxacin | Fluoroquinolone-class; inhibits DNA gyrase | 0.10 | 0.99 |
|  | DK-507K | Fluoroquinolone-class; inhibits DNA gyrase | 0.11 | 0.99 |
|  | PD-131112 | Fluoroquinolone-class; inhibits DNA gyrase | 0.12 | 0.99 |
|  | E-4884 | Fluoroquinolone-class; inhibits DNA gyrase | 0.12 | 0.99 |
|  | Sparfloxacin | Fluoroquinolone-class; inhibits DNA gyrase | 0.13 | 0.98 |
|  | Clinafloxacin | Fluoroquinolone-class; inhibits DNA gyrase | 0.15 | 0.99 |
|  | BMY-43748 | Fluoroquinolone-class; inhibits DNA gyrase | 0.15 | 0.99 |
|  | R-71762 | Fluoroquinolone-class; inhibits DNA gyrase | 0.17 | 0.99 |
|  | Danofloxacin | Fluoroquinolone-class; inhibits DNA gyrase | 0.17 | 0.99 |
|  | Moxifloxacin | Fluoroquinolone-class; inhibits DNA gyrase | 0.17 | 0.99 |
|  | Finafloxacin | Fluoroquinolone-class; inhibits DNA gyrase | 0.18 | 0.99 |
|  | Grepafloxacin | Fluoroquinolone-class; inhibits DNA gyrase | 0.19 | 0.99 |
|  | WQ-2756 | Fluoroquinolone-class; inhibits DNA gyrase | 0.19 | 0.97 |
|  | DC-159a | Fluoroquinolone-class; inhibits DNA gyrase | 0.20 | 0.99 |
|  | Difloxacin | Fluoroquinolone-class; inhibits DNA gyrase | 0.20 | 0.99 |
|  | Flumequine | Fluoroquinolone-class; inhibits DNA gyrase | 0.21 | 0.99 |
|  | Cadrofloxacin | Fluoroquinolone-class; inhibits DNA gyrase | 0.21 | 0.99 |
|  | E-4904 | Fluoroquinolone-class; inhibits DNA gyrase | 0.21 | 0.99 |
|  | KPI-10 | Fluoroquinolone-class; inhibits DNA gyrase | 0.22 | 0.97 |
|  | E-5065 | Fluoroquinolone-class; inhibits DNA gyrase | 0.24 | 0.99 |
|  | BMY-41802 | Fluoroquinolone-class; inhibits DNA gyrase | 0.25 | 0.99 |
|  | Levofloxacin | Fluoroquinolone-class; inhibits DNA gyrase | 0.28 | 0.99 |
|  | PD-160793 | Fluoroquinolone-class; inhibits DNA gyrase | 0.31 | 0.98 |
|  | Temafoxacin | Fluoroquinolone-class; inhibits DNA gyrase | 0.31 | 0.98 |
|  | Y-688 | Fluoroquinolone-class; inhibits DNA gyrase | 0.31 | 0.98 |
|  | Gemifloxacin | Fluoroquinolone-class; inhibits DNA gyrase | 0.34 | 0.99 |
|  | Gatifloxacin | Fluoroquinolone-class; inhibits DNA gyrase | 0.34 | 0.97 |
|  | DV-7751A | Fluoroquinolone-class; inhibits DNA gyrase | 0.45 | 0.99 |
|  | Pefloxacin | Fluoroquinolone-class; inhibits DNA gyrase | 0.46 | 0.99 |
|  | Pazufloxacin | Fluoroquinolone-class; inhibits DNA gyrase | 0.49 | 0.96 |
|  | Marbofloxacin | Fluoroquinolone-class; inhibits DNA gyrase | 0.69 | 0.96 |
|  | RFM-003-154-9 | Fluoroquinolone-class; inhibits DNA gyrase | 0.61 | 0.99 |
|  | WCK-1152 | Fluoroquinolone-class; inhibits DNA gyrase | 0.71 | 0.99 |
|  | DW-286 | Fluoroquinolone-class; inhibits DNA gyrase | 0.72 | 0.99 |
|  | Besifloxacin | Fluoroquinolone-class; inhibits DNA gyrase | 0.87 | 0.91 |
|  | Lomefloxacin | Fluoroquinolone-class; inhibits DNA gyrase | 0.91 | 0.94 |
|  | CP-74667 | Fluoroquinolone-class; inhibits DNA gyrase | 1.09 | 0.99 |
|  | Protosufloxacin | Fluoroquinolone-class; inhibits DNA gyrase | 1.20 | 0.99 |
|  | Sarafloxacin | Fluoroquinolone-class; inhibits DNA gyrase | 1.31 | 0.97 |
|  | Ulifloxacin | Fluoroquinolone-class; inhibits DNA gyrase | 2.06 | 0.99 |
|  | Ecenofloxacin | Fluoroquinolone-class; inhibits DNA gyrase | 2.41 | 0.97 |
|  | OPC-7241 (Vebufloxacin) | Fluoroquinolone-class; inhibits DNA gyrase | 2.79 | 0.92 |
|  | Rufloxacin | Fluoroquinolone-class; inhibits DNA gyrase | 3.59 | 0.99 |
|  | Irloxacin | Quinolone-class; inhibits DNA gyrase | 0.15 | 0.97 |
|  | DJ-6783 | Quinolone-class; inhibits DNA gyrase | 0.18 | 0.99 |
|  | Rosoxacin | Quinolone-class; inhibits DNA gyrase | 0.18 | 0.98 |
|  | PD-161314 | Quinolone-class; inhibits DNA gyrase | 0.22 | 0.99 |
|  | Garenoxacin | Quinolone-class; inhibits DNA gyrase | 0.44 | 0.98 |
|  | Nemonoxacin | Quinolone-class; inhibits DNA gyrase | 0.49 | 0.98 |
|  | PGE-9509924 | Quinolone-class; inhibits DNA gyrase | 0.56 | 0.99 |
|  | Nitroxoline | Quinolone-class; inhibits DNA gyrase | 9.63 | 0.88 |
| **Host-directed compounds** | AQW-051 | Agonist of 𝝰7 neuronal nicotinic ACh receptor expressed in CNS; receptor also expressed in tissue-resident macrophages and involved in cholinergic anti-inflammatory responses | 2.46 | 0.97 |
|  | PD-122655 | Adrenergic Receptor Alpha-2 Antagonist | 2.55 | 0.99 |

^a^Compounds were resupplied in 8-point dose response format with concentrations ranging from 0.00467 to 9.99 µM and tested on 2 separate occasions; average EC_50_ values are shown in µM. Compounds with average EC_50_ values >9.99 µM were considered unconfirmed and are not listed.

^b^Average R^2^ values are shown.
