# Supplementary material for: Discovery of repurposed drugs that disrupt intracellular replication of Burkholderia pseudomallei and Burkholderia mallei and protect against lethal aerosol infection
Source: PLoS Pathog. 2026 Sep 16;22(9):e1014596. doi: 10.1371/journal.ppat.1014596 (PMC13600621; doi:10.1371/journal.ppat.1014596)
Supplement: Supplementary file 5 [file ppat.1014596.s005.docx]

**S5 Table. Plate metrics for ReFRAME single-point screen using *Burkholderia mallei* strain ATCC 23344.**

| Plate | PAS Z′-factor | Mean PAS TMP-treated controls (n =16 wells per plate) | Mean PAS untreated controls (n =16 wells per plate) |
| --- | --- | --- | --- |
| W1026439 | 0.72 | 1.90E+06 ± 191E+03 | 8.94E+06 ± 472E+03 |
| W1026441 | 0.51 | 1.80E+06 ± 97.2E+03 | 7.94E+06 ± 899E+03 |
| W1026443 | 0.61 | 1.89E+06 ± 113E+03 | 8.22E+06 ± 702E+03 |
| W1026445 | 0.76 | 1.86E+06 ± 209E+03 | 9.05E+06 ± 369E+03 |
| W1026447 | 0.73 | 2.05E+06 ± 232E+03 | 9.35E+06 ± 433E+03 |
| W1026449 | 0.66 | 2.00E+06 ± 163E+03 | 9.11E+06 ± 637E+03 |
| W1026451 | 0.48 | 2.05E+06 ± 187E+03 | 8.01E+06 ± 842E+03 |
| W1026453 | 0.22 | 2.19E+06 ± 252E+03 | 8.08E+06 ± 1.28E+06 |
| W1026455 | 0.61 | 1.77E+06 ± 118E+03 | 7.74E+06 ± 667E+03 |
| W1026457 | 0.64 | 2.01E+06 ± 146E+03 | 8.77E+06 ± 667E+03 |
| W1026459 | 0.4 | 1.99E+06 ± 139E+03 | 7.15E+06 ± 889E+03 |
| W1026461 | 0.58 | 2.11E+06 ± 286E+03 | 8.54E+06 ± 609E+03 |
| W1026463 | 0.56 | 1.92E+06 ± 121E+03 | 8.58E+06 ± 851E+03 |
| W1026465 | 0.66 | 2.31E+06 ± 350E+03 | 9.15E+06 ± 437E+03 |
| W1026467 | 0.73 | 2.09E+06 ± 204E+03 | 9.55E+06 ± 471E+03 |
| W1026469 | 0.53 | 1.90E+06 ± 235E+03 | 8.01E+06 ± 715E+03 |
| W1026471 | 0.61 | 2.27E+06 ± 203E+03 | 9.20E+06 ± 693E+03 |
| W1026473 | 0.72 | 2.29E+06 ± 304E+03 | 9.62E+06 ± 384E+03 |
| W1026475 | 0.46 | 1.82E+06 ± 81.0E+03 | 7.39E+06 ± 919E+03 |
| W1026477 | -0.18 | 2.54E+06 ± 636E+03 | 7.79E+06 ± 1.43E+06 |
| W1026479 | 0.52 | 2.17E+06 ± 458E+03 | 9.57E+06 ± 714E+03 |
| W1026481 | 0.65 | 2.03E+06 ± 296E+03 | 9.54E+06 ± 572E+03 |
| W1026483 | 0.76 | 2.30E+06 ± 238E+03 | 9.96E+06 ± 376E+03 |
| W1026485 | 0.65 | 2.59E+06 ± 429E+03 | 9.99E+06 ± 438E+03 |
| W1026487 | 0.76 | 1.98E+06 ± 144E+03 | 9.64E+06 ± 481E+03 |
| W1026489 | 0.65 | 1.98E+06 ± 137E+03 | 9.55E+06 ± 740E+03 |
| W1026491 | 0.58 | 1.63E+06 ± 123E+03 | 9.07E+06 ± 922E+03 |
| W1026493 | 0.77 | 1.86E+06 ± 263E+03 | 9.72E+06 ± 335E+03 |
| W1026495 | 0.77 | 1.77E+06 ± 242E+03 | 10.0E+06 ± 401E+03 |
| W1026497 | 0.67 | 1.89E+06 ± 146E+03 | 8.68E+06 ± 599E+03 |
| W1026499 | 0.78 | 1.81E+06 ± 176E+03 | 10.1E+06 ± 429E+03 |
| W1026501 | 0.7 | 1.73E+06 ± 188E+03 | 9.30E+06 ± 568E+03 |
| W1026503 | 0.59 | 1.87E+06 ± 162E+03 | 7.49E+06 ± 598E+03 |
| W1026505 | 0.72 | 1.76E+06 ± 169E+03 | 9.08E+06 ± 505E+03 |
| W1026507 | 0.81 | 1.84E+06 ± 196E+03 | 9.58E+06 ± 291E+03 |
| W1026509 | 0.47 | 1.98E+06 ± 605E+03 | 9.05E+06 ± 655E+03 |
| W1026511 | 0.72 | 2.01E+06 ± 194E+03 | 9.75E+06 ± 520E+03 |
| W1026513 | 0.84 | 1.82E+06 ± 174E+03 | 10.3E+06 ± 284E+03 |
| W1026515 | 0.8 | 2.35E+06 ± 201E+03 | 10.9E+06 ± 375E+03 |

PAS = plaque area sum (µm^2^); mean is presented as mean PAS ± standard deviation; collective (n = 39 plates) mean Z’-factor 0.62; collective (n = 39 plates) mean PAS TMP-treated controls 1.90E+6 ± 2.27E+05; collective (n = 39 plates) mean PAS untreated controls 8.96E+06 ± 6.26E+05
