# Supplementary material for: Discovery of repurposed drugs that disrupt intracellular replication of Burkholderia pseudomallei and Burkholderia mallei and protect against lethal aerosol infection
Source: PLoS Pathog. 2026 Sep 16;22(9):e1014596. doi: 10.1371/journal.ppat.1014596 (PMC13600621; doi:10.1371/journal.ppat.1014596)
Supplement: Supplementary file 6 [file ppat.1014596.s006.docx]

**S6 Table. EC_50_ values of ReFRAME hits protecting against intracellular replication of *Burkholderia mallei* ATCC23344.**

| **Compound type** | **Compound name** | **Mechanism of Action** | **^a^EC_50_** | **^b^R^2^** |
| --- | --- | --- | --- | --- |
| **Antibiotics interfering with DNA synthesis** | Iclaprim | Inhibits dihydropholate reductase (DHFR) | 0.05 | 0.99 |
|  | Brodimoprim | Inhibits dihydropholate reductase (DHFR) | 0.06 | 0.98 |
|  | 1954U89 | Inhibits dihydropholate reductase (DHFR) | 0.11 | 0.99 |
|  | Trimethoprim (TMP) | Inhibits dihydropholate reductase (DHFR) | 0.12 | 0.90 |
|  | Ormetoprim | Inhibits dihydropholate reductase (DHFR) | 0.36 | 0.98 |
|  | Aditoprim | Inhibits dihydropholate reductase (DHFR) | 0.44 | 0.99 |
|  | Metioprim | Inhibits dihydropholate reductase (DHFR | 0.64 | 0.92 |
|  | 2,4-Diamino-5-(3,4-Dimethoxybenzyl) Pyrimidine | Inhibits dihydropholate reductase (DHFR) | 0.73 | 0.97 |
|  | Piritrexim | Inhibits dihydropholate reductase (DHFR) | 0.88 | 0.94 |
|  | Baquiloprim | Inhibits dihydropholate reductase (DHFR) | 1.00 | 0.95 |
|  | DAMP-ES | Inhibits dihydropholate reductase (DHFR) | 5.77 | 0.99 |
|  | RFM-003-805-1 | Sulfonamide-class; inhibits dihydropteroate synthase (DHPS) | 0.28 | 0.97 |
|  | Chlorosulfaquinoxaline | Sulfonamide-class; inhibits dihydropteroate synthase (DHPS) | 5.57 | 0.99 |
|  | 5-fluorouracil | Inhibits thymidylate synthase and the production of deoxythymidine monophosphate | 2.69 | 0.99 |
|  | Azanidazole | Nitroimidazole-class; causes DNA damage | 5.74 | 0.94 |
| **Antibiotics interfering with protein synthesis** | Nitrocycline | Tetracycline-class; binds 30S subunit of bacterial ribosome and hinders binding of tRNA | 0.02 | 0.91 |
|  | Eravacycline | Tetracycline-class; binds 30S subunit of bacterial ribosome and hinders binding of tRNA | 0.03 | 0.96 |
|  | Meclocycline | Tetracycline-class; binds 30S subunit of bacterial ribosome and hinders binding of tRNA | 0.03 | 0.96 |
|  | Doxycycline | Tetracycline-class; binds 30S subunit of bacterial ribosome and hinders binding of tRNA | 0.03 | 0.97 |
|  | Minocycline | Tetracycline-class; binds 30S subunit of bacterial ribosome and hinders binding of tRNA | 0.04 | 0.98 |
|  | Demeclocycline | Tetracycline-class; binds 30S subunit of bacterial ribosome and hinders binding of tRNA | 0.06 | 0.92 |
|  | Methacycline | Tetracycline-class; binds 30S subunit of bacterial ribosome and hinders binding of tRNA | 0.07 | 0.86 |
|  | RFM-001-392-3 | Tetracycline-class; binds 30S subunit of bacterial ribosome and hinders binding of tRNA | 0.13 | 0.88 |
|  | Omadacycline | Tetracycline-class; binds 30S subunit of bacterial ribosome and hinders binding of tRNA | 0.19 | 0.96 |
|  | Incyclinide | Tetracycline-class; binds 30S subunit of bacterial ribosome and hinders binding of tRNA | 0.38 | 0.94 |
|  | Chlortetracycline | Tetracycline-class; binds 30S subunit of bacterial ribosome and hinders binding of tRNA | 3.01 | 0.99 |
|  | Chloramphenicol | Chloramphenicol-class; binds 50S subunit of bacterial ribosome and inhibits the action of peptidyl transferase | 0.92 | 0.98 |
|  | Valnemulin | Pleuromutilin-class; binds 50S subunit of bacterial ribosome and inhibits the action of peptidyl transferase | 5.72 | 0.91 |
|  | VRC-4411 | Inhibits activity of bacterial peptide deformylase (PDF) | 0.38 | 0.99 |
|  | GSK-1322322 | Inhibits activity of bacterial peptide deformylase (PDF) | 0.52 | 0.97 |
|  | GBB-83698 | Inhibits activity of bacterial peptide deformylase (PDF) | 1.15 | 0.96 |
|  | Sodium fusidate | Prevents turnover of elongation factor G from the bacterial ribosome | 3.17 | 0.98 |
|  | Epetraborole | Benzoxaborole-class; boron-heterocyclic compound that targets bacterial leucyl-tRNA synthase (LeuRS) | 0.36 | 0.98 |
| **Antibiotics interfering with fatty acid biosynthesis** | Debio 1452 (API-1252) | Inhibits activity of bacterial FabI enoyl-acyl carrier protein (ACP) reductase | 0.86 | 0.98 |
| **Antibiotics interfering with RNA synthesis** | Mycobutin | Rifamycin-class; binds 𝛃 subunit of bacterial RNA polymerase and inhibits synthesis of RNA | 0.37 | 0.99 |
|  | LM-565 | Rifamycin-class; binds 𝛃 subunit of bacterial RNA polymerase and inhibits synthesis of RNA | 0.40 | 0.99 |
|  | Rifapentine | Rifamycin-class; binds 𝛃 subunit of bacterial RNA polymerase and inhibits synthesis of RNA | 1.05 | 0.99 |
| **Antibiotics interfering with synthesis of lipopolysaccharides** | ACHN-975 | Inhibits activity of bacterial Uridine diphosphate-3-O-(hydroxymyristoyl)-N-acetylglucosamine deacetylase LpxC in Gram-negative bacteria | 2.73 | 0.96 |
| **Antibiotics interfering with DNA replication** | AZD-9742 | Fluoroquinolone-class; inhibits DNA gyrase | 0.43 | 0.99 |
|  | A-83669 | Fluoroquinolone-class; inhibits DNA gyrase | 0.93 | 0.98 |
|  | AZD-0914 (Zoliflodacin) | Fluoroquinolone-class; inhibits DNA gyrase | 1.32 | 0.99 |
|  | A-84441 | Fluoroquinolone-class; inhibits DNA gyrase | 5.76 | 0.98 |
|  | DJ-6783 | Quinolone-class; inhibits DNA gyrase | 0.12 | 0.99 |
| **Antibiotics interfering with bacterial physiology** | Bismuth ethanedithiol | Disrupts biofilm formation; interferes with iron transport and redox enzymes involved in extracellular polysaccharides expression via thiol exchange | 9.95 | 0.89 |
| **Host-directed compounds** | Phorbol 12-myristate 13-acetate | Protein kinase C activator; stimulates T-cell activation and cytokine production | 0.01 | 0.74 |
|  | Lofemizole | Non-steroidal anti-inflammatory agent | 0.94 | 0.94 |
|  | PD-122655 | Adrenergic Receptor Alpha-2 Antagonist | 0.71 | 0.99 |
|  | GK 128 | 5-hydroxytryptamine (5-HT)_3_ receptor antagonist | 1.06 | 0.97 |
|  | GT-2314 | High affinity histamine H3 receptor antagonist | 2.82 | 0.92 |
|  | MCI-727 | Anti-ulcer agent; increases production of secretin | 5.53 | 0.96 |
|  | Vesnarinone | Inotropic agent; inhibits activity of phosphodiesterase III enzyme | 5.94 | 0.98 |
|  | Serdemetan | Anti-cancer drug that inhibits the binding of the HDM2 protein to the transcriptional activation domain of p53 | 5.97 | 0.97 |
|  | GT-2016 | Selective and brain penetrant histamine H3 receptor antagonist; displays selectivity against H1 and H2 receptors | 6.31 | 0.98 |
|  | Ingenol mebutate | Protein kinase C activator; causes cell death via necrosis | 9.95 | 0.94 |
|  | AQW-051 | Agonist of 𝝰7 neuronal nicotinic ACh receptor expressed in CNS; receptor also expressed in tissue-resident macrophages and involved in cholinergic anti-inflammatory responses | 9.95 | 0.97 |
| **Not disclosed due to proprietary restrictions** | CBR-001-584-732-2 | Not available | 0.06 | 0.94 |
|  | CBR-001-601-505-7 | Not available | 0.09 | 0.98 |
|  | CBR-001-622-664-5 | Not available | 0.48 | 0.99 |
|  | CBR-001-585-236-5 | Not available | 0.56 | 0.99 |
|  | CBR-001-669-920-0 | Not available | 0.63 | 0.98 |
|  | CBR-001-670-841-1 | Not available | 1.12 | 0.99 |
|  | CBR-001-624-900-6 | Not available | 1.16 | 0.97 |
|  | CBR-001-670-845-5 | Not available | 1.59 | 0.79 |
|  | CBR-001-586-354-4 | Not available | 1.83 | 0.99 |
|  | CBR-001-585-170-4 | Not available | 2.17 | 0.99 |
|  | CBR-001-587-102-0 | Not available | 2.53 | 0.99 |
|  | CBR-001-841-997-1 | Not available | 3.01 | 0.99 |
|  | CBR-001-573-388-7 | Not available | 4.99 | 0.97 |
|  | CBR-001-670-518-3 | Not available | 5.63 | 0.99 |
|  | CBR-001-571-351-6 | Not available | 5.82 | 0.96 |
|  | CBR-001-598-993-2 | Not available | 5.86 | 0.82 |
|  | CBR-001-598-981-8 | Not available | 6.02 | 0.99 |
|  | CBR-001-585-385-7 | Not available | 9.95 | 0.45 |

^a^Compounds were resupplied in 8-point dose response format with concentrations ranging from 0.004521 to 10 µM and tested on 2 separate occasions; average EC_50_ values are shown in µM. Compounds with EC_50_ values ≥ 9.99 µM were considered unconfirmed and are not listed.

^b^Average R^2^ values are shown.
